# Development of Chatbot-Based Oral Health Care for Young Children and Evaluation of its Effectiveness, Usability, and Acceptability: Mixed Methods Study

**DOI:** 10.2196/62738

**Published:** 2025-02-03

**Authors:** Kittiwara Pupong, Jaranya Hunsrisakhun, Samerchit Pithpornchaiyakul, Supawadee Naorungroj

**Affiliations:** 1 Dental Public Health Division, Maelan Hospital, Pattani, Thailand; 2 Department of Preventive Dentistry, Faculty of Dentistry, Prince of Songkla University, 15 Kanjanavanich Rd, Hatyai, Songkhla, 90112, Thailand, 66 74429875, 66 74429875; 3 Improvement of Oral Health Care Research Unit, Faculty of Dentistry, Prince of Songkla University, Hatyai, Songkhla, Thailand; 4 Department of Conservative Dentistry, Faculty of Dentistry, Prince of Songkla University, Hatyai, Songkhla, Thailand

**Keywords:** chatbot, conversational agents, tele-dentistry, oral health behavior, in-person toothbrushing, hands-on, children, covid-19, oral health education, development.

## Abstract

**Background:**

Chatbots are increasingly accepted in public health for their ability to replicate human-like communication and provide scalable, 24/7 services. The high prevalence of dental caries in children underscores the need for early and effective intervention.

**Objective:**

This study aimed to develop the 30-Day FunDee chatbot and evaluate its effectiveness, usability, and acceptability in delivering oral health education to caregivers of children aged 6 to 36 months.

**Methods:**

The chatbot was created using the artificial intelligence (AI) chatbot behavior change model, integrating behavioral change theories into content designed for 3‐5 minutes of daily use over 30 days. A pre-post experimental study was conducted from December 2021 to February 2022 in Hat Yai District, Songkhla Province, and Maelan District, Pattani Province, Thailand. Fifty-eight caregivers completed a web-based structured questionnaire at baseline and 2 months post baseline to evaluate knowledge, protection motivation theory-based perceptions, and tooth-brushing practices. Usability was assessed via chatbot logfiles and a web-based questionnaire at 2 months post baseline. Acceptability was evaluated through three methods: (1) open-ended chatbot interactions on day 30, (2) a web-based structured questionnaire at 2 months post baseline, and (3) semistructured telephone interviews with 15 participants 2 weeks post intervention. Participants for interviews were stratified by adherence levels and randomly selected from Hatyai and Maelan districts. All self-reported variables were measured on a 5-point Likert scale (1=lowest, 5=highest).

**Results:**

The chatbot was successfully developed based on the 4 components of the AI chatbot behavior change model. Participants had a mean age of 34.5 (SD 8.6) years. The frequency of tooth brushing among caregivers significantly improved, increasing from 72.4% at baseline to 93.1% two months post baseline (*P*=.006). Protection motivation theory-based perceptions also showed significant improvement, with mean scores rising from 4.0 (SD 0.6) at baseline to 4.5 (SD 0.6) two months post baseline (*P*<.001). The chatbot received high ratings for satisfaction (4.7/5, SD 0.6) and usability (4.7/5, SD 0.5). Participants engaged with the chatbot for an average of 24.7 (SD 7.2) days out of 30. Caregivers praised the chatbot’s content quality, empathetic communication, and multimedia design, but noted the intervention’s lengthy duration and messaging system as limitations.

**Conclusions:**

The 30-Day FunDee chatbot effectively enhanced caregivers’ perceptions of oral health care and improved tooth-brushing practices for children aged 6‐36 months. High user satisfaction and engagement demonstrate its potential as an innovative tool for oral health education. These findings warrant further validation through large-scale, randomized controlled trials.

## Introduction

Chatbots have emerged as a socially responsible technology to bridge socioeconomic disparities and promote equitable access to high-quality health care [1,2]. Chatbots are digital tools that emulate human conversation and have the potential to promote health education, support behavior changes, and deliver health care, especially for clinical or vulnerable populations. Their adaptability also makes them suitable for deployment in a variety of sizeable and diverse samples [3]. Chatbots provide flexible, on-demand support, offering personalized assistance and content with continuous availability, helping to mitigate the limitations of traditional telehealth services [4]. Overall, chatbots offer a humanized interaction that can support health care professionals in managing and preventing conditions on a societal scale [5].

Multiple studies attest to the effectiveness of incorporating chatbots in health care, illustrating their role in facilitating a modification of health-related behaviors [3,6-8]. However, the existing literature on the use of chatbots for dental health purposes is limited [9,10], and few studies have explored the complicated process of constructing chatbots with oral health goals in detail.

Zhang et al [11] suggested some guidelines for the development of an artificial intelligence (AI) chatbot for behavior modification with the formulation of a theoretical framework that consists of four essential components: (1) specifying chatbot characteristics and understanding user backgrounds, (2) establishing relational capacity, (3) establishing persuasive capacity using behavioral-based theory, and (4) implementing an evaluation mechanism to assess outcomes.

Early childhood caries (ECC), identified as a significant chronic disease, emerges from the dynamic interplay of various risk and protective factors over time [12]. In Thailand, the earliest detection of noncavitated caries occurred at 9 months of age, while cavitation was first identified at 10 months of age [13]. In this context, parental tooth brushing, conducted twice daily with a rice-sized amount of fluoridated toothpaste, has been proven to be a pivotal strategy for reducing dental caries in children [12], whereas systematic reviews revealed that the effectiveness of simple health education in preventing ECC is restricted [14]. In line with a review regarding oral health promotion for ECC, home visits, and hands-on techniques for dental health education substantially enhance habit formation and effectiveness [15]. In addition, caregivers should receive practical training and empowerment from oral health professionals [16]. However, these interventions must be tailored to each individual, requiring not only highly skilled dental personnel but also considerable time and financial resources [17]. Chatbots have the potential to serve as a scalable model for skill development, motivation, problem-solving, and continuous follow-up, replicating the advantages of home visits.

Habits emerge as a consequence of acquiring repeated patterns of behavior, originating from consistent contextual stimuli, and they are formed through automated responses to the conditions in which they happen [18]. The research findings revealed that the time required to form habits varied, with values ranging from 18 to 254 days, depending on the level of complexity or challenge associated with a particular behavior [19]. Monitoring and reinforcement are effective ways to develop healthy habits [19,20] toward caries prevention in children [15], however, interaction with health professionals often requires traditional onsite visits, substantial time, and financial cost [21,22].

The protection motivation theory (PMT), a key theory in health psychology, explains how people change their behavior based on their evaluation of threats and their ability to cope with them [23,24]. The threat appraisal component of the PMT comprises 2 key elements: the individual’s assessment of the disease’s severity (perceived severity) and the probability of developing the disease (perceived vulnerability). The PMT additionally specifies that attitudes and behavior modification are indirectly influenced by the emotional state of fear arousal, which occurs through the evaluation of the danger’s severity. The coping appraisal of the model comprises the individual’s response efficacy, which is the expectation that implementing the recommendations will eliminate the threat, and self-efficacy, which is the belief in one’s own ability to effectively execute the recommended course of action. Behavior performance should be significantly influenced by the intentions that are mediated by the cognitive predictors of threat and coping appraisals [25,26]. Kimhasawad et al [27] used a PMT-based education program to guide caregivers of 9‐ to 12-month-old infants in adopting proper tooth brushing techniques, revealing that this approach effectively motivated and increased awareness, leading to a positive change in the oral health behavior of caregivers.

Currently, there is no standard evaluation method for a health care chatbot [28,29]. The concepts of usability and acceptability were identified as important factors for evaluating the use of chatbots [17]. However, there are differing definitions and some overlap between these terms [29,30]. Our study adopted broad interpretations of these concepts: usability as use, engagement, and ease of use; acceptability as satisfaction; continued use intention; and appropriateness. In addition, the frameworks of Zhang et al [11] and Denecke et al [31] were applied for content evaluation integrating usability and acceptability. This comprehensive evaluation provides a holistic assessment of the factors influencing chatbot adoption [32].

Therefore, the study aimed to elucidate the chatbot development process using PMT and Zhang’s model. In addition, the pre-post study sought to assess the effectiveness, usability, and acceptability of a developed chatbot, the “30-Day FunDee,” which was intended to educate caregivers of children between 6 and 36 months regarding oral health.

## Methods

**Figure 1.**
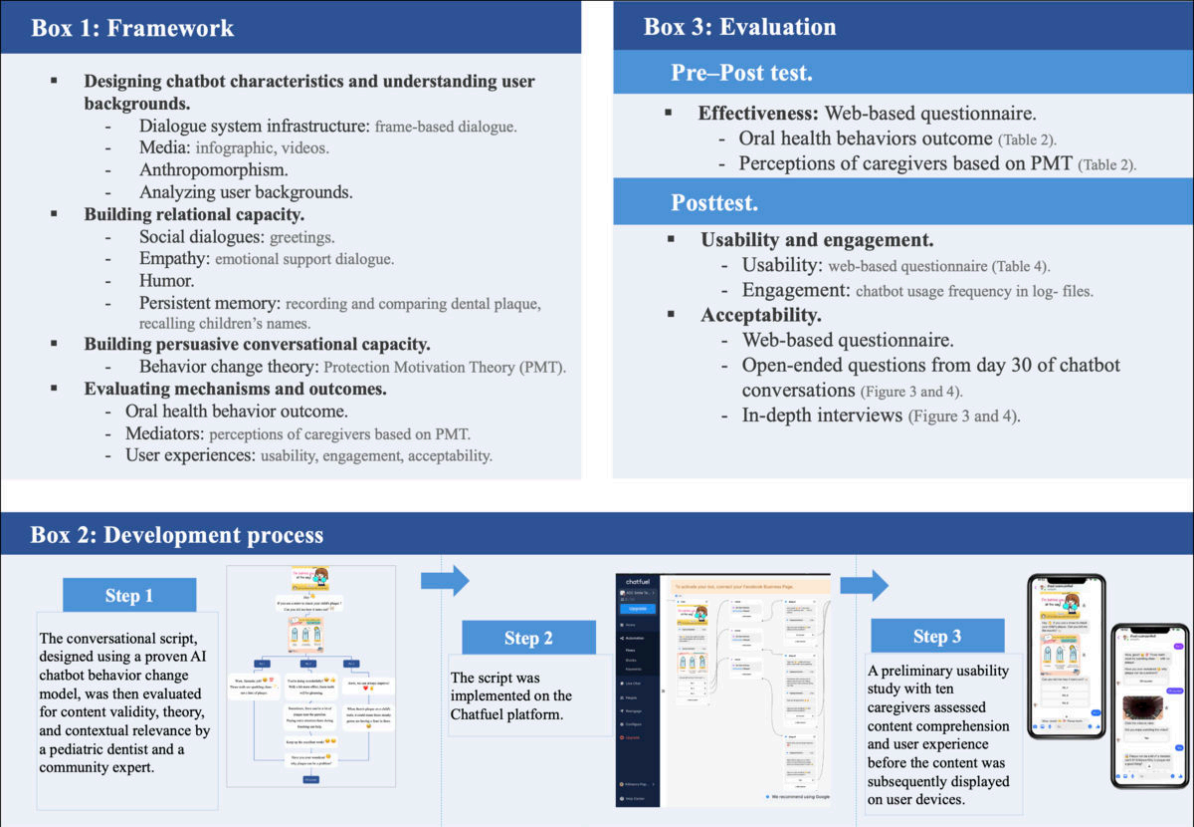
Framework, development, and evaluation of the 30-day FunDee chatbot. AI: artificial intelligence.

Figure 1 illustrates the comprehensive process involved in the defining framework, development, and evaluation of the 30-Day FunDee oral health promotion chatbot.

### Defining a Framework for the 30-Day FunDee Chatbot

#### Designing Chatbot Characteristics and Understanding User Background

**Figure 2.**
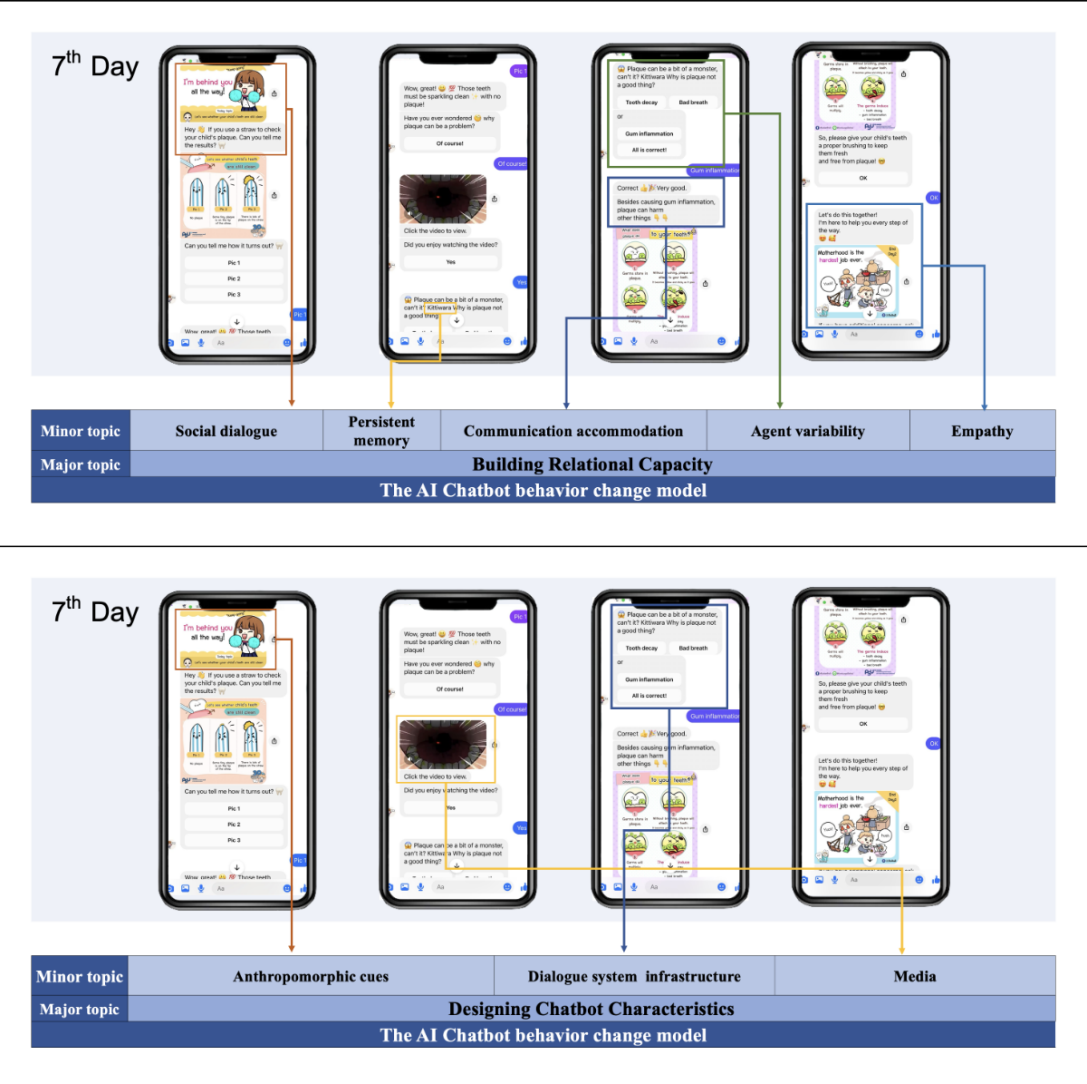
Screen example of the chatbot translated from Thai to English, showing dialogues that incorporate the design characteristics of the AI behavior change model on the seventh day. AI: artificial intelligence.

The objectives and target audience were specified for educating and assisting Thai caregivers with oral hygiene care for children aged 0 to 3 years. Designing chatbot characteristics encompassed dialogue system infrastructure, media, and anthropomorphism [11] as shown in Figure 2. The chatbot’s dialogue system infrastructure is frame-based, enabling users to select their responses. The chatbot created a variety of media, including simple and clear language, illustrations, written text, audio components, conversational dialogue, infographics, animations, animated songs, and videos with authentic and animated content. Moreover, within the anthropomorphic realm, “Dr. Pin” the chatbot’s dentist avatar, was depicted as a kind, charming, empathetic, and attentive caregiver for children’s oral health.

Understanding the user’s background is critical in designing chatbots for behavior change [11,33]. Based on previous studies [34-36], the majority of caregivers were 20- to 40-year-old mothers with low to middle socioeconomic status who were responsible for nurturing their children, especially in relation to their children’s tooth brushing. The chatbot’s role was to assist users in overcoming obstacles and offering emotional support in childcare. Due to internet availability, both urban and rural caregivers could access the chatbot.

The content of the 30-Day FunDee chatbot was influenced by pediatric and community dentistry experts’ recommendations and qualitative research insights on Thai caregivers’ experiences with children’s tooth-brushing behaviors [35,36], insights from community caregivers, inquiries from dental clinics, and knowledge gained from the earlier 21-Day FunDee chatbot, which is mentioned elsewhere [9]. Therefore, the updated chatbot was extended from 21 to 30 days, including more various media and dialogues, especially focusing on tooth-brushing practices.

#### Building Relational Capacity

Building relational capacity involves using elements such as social dialogues, empathy, relationship discussion, humor, self-disclosure, persistent memory, and agent variability [11,29]. Daily, the chatbot initiated conversations with charming greetings and concluded with farewell messages (Figure 2) that conveyed understanding and encouraged parents or caregivers in childcare [11]. For goal setting [37], daily welcome cards were designed with topics to be discussed.

The chatbot was designed to consistently display comprehension, empathy, and emotional support during interactions, especially when users encountered obstacles. Emojis and animated GIFs conveying positive feedback and emotions were incorporated to increase engagement. Progress tracking enabled self-comparison and confidence-building over time. Periodic child dental plaque assessments prompted reflection on tooth-brushing techniques. Positive reinforcement was provided when plaque decreased, while setbacks received encouragement without punishment. Conversation features like remembering children’s names, revisiting prior discussions, and using age-appropriate informal language aimed to simulate natural human-to-human dialogues. These elements enhanced the user experience and facilitated task completion [37,38] (Figure 2).

#### Building Persuasive Conversational Capacity

##### Overview

**Figure 3.**
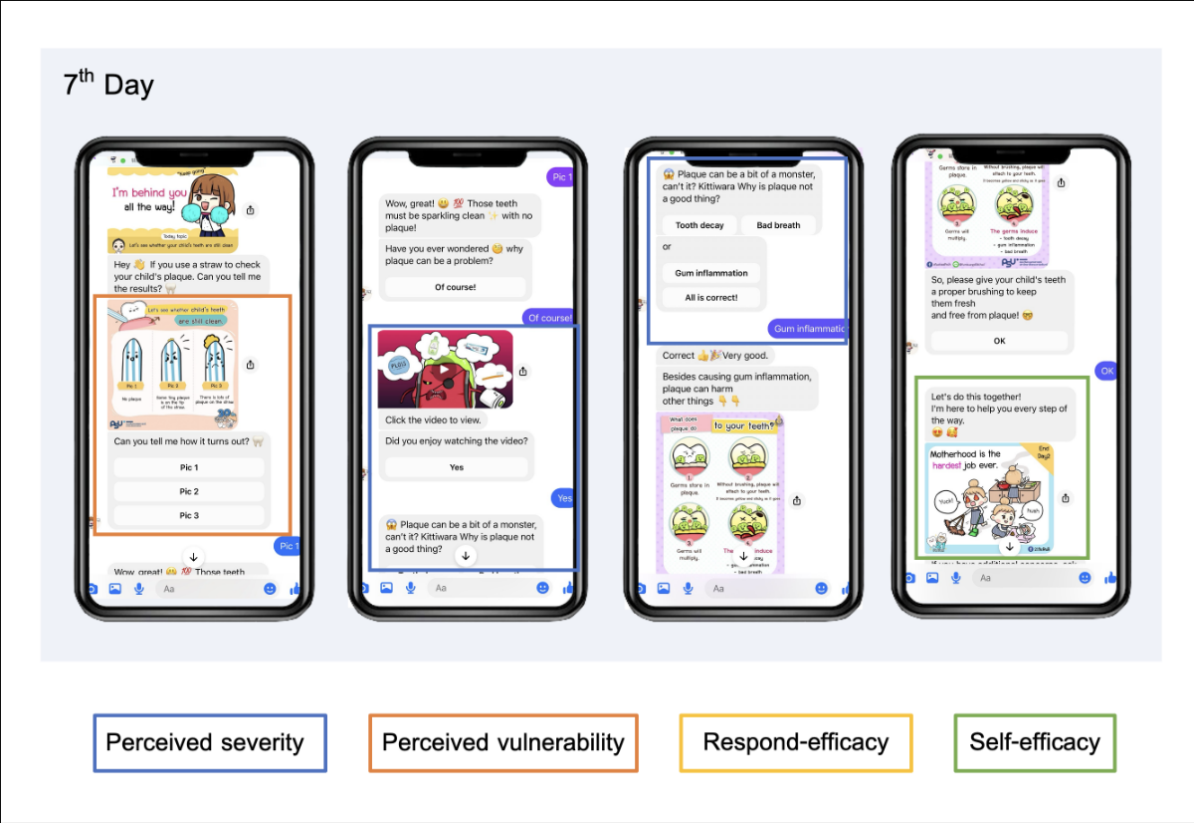
Screen examples of chatbot dialogues translated from Thai to English, incorporating protection motivation theory (PMT) on the seventh day.

The 30-Day FunDee chatbot, meticulously designed, provided oral health education to caregivers based on PMT (Figure 3) for behavior modification by systematically sequencing threats and coping topics. Examples of chatbot topics are shown in the following sections.

##### Perceived Severity

The importance of primary teeth, the consequences of dental infections, and anticipating problems from poor oral health care were discussed.

##### Perceived Vulnerability

The chatbot instructed caregivers on the child’s dental plaque assessment and provided animation and infographics explaining plaque. Periodic plaque checks were encouraged, and an API enabled tracking of plaque levels over time.

##### Belief in Response Efficacy

Benefits and instructions, especially for tooth brushing, were incorporated into a variety of videos, infographics, and games. The chatbot also asked caregivers about children’s sugar intake and diet to discuss potential health impacts.

##### Belief in Self-Efficacy

The chatbot was designed to increase caregiver self-efficacy for children’s oral health management through motivational techniques and daily emotional support. At the end of each conversation, it expressed empathy for mothers’ exhaustion and encouraged overcoming obstacles.

### Chatbot Development Process

The development of chatbots followed a systematic, 3-phase process, as illustrated in Figure 1. The first step involved designing the conversational flow, which included scripts, infographics, videos, and other content. This step was crucial to ensure that the chatbot can engage effectively with users. This phase followed an established AI chatbot behavior change model and PMT, which guided the creation of responsive conversations tailored to encourage specific behavioral outcomes. The next step involved implementing the conversational flow using the Chatfuel platform, which enabled the transformation of the predesigned scripts into interactive conversations accessible on users’ devices. The Chatfuel platform facilitated the efficient deployment of the chatbot design, ensuring seamless operation and scalability to accommodate the intended target audience, without advanced programming skills and includes push message functionality [39]. Given that over 80% of Thailand’s population uses Facebook [40], Facebook Messenger was chosen as the delivery platform using Chatfuel.

The final step involved presenting the chatbot’s content on user devices. After implementation, the chatbot became accessible, with its content displayed across various devices. This stage completed the chatbot’s development, enabling it to interact with users as intended and deliver the conversations crafted during the initial phase. The chatbot used persuasive communication, including comprehension, empathy, and suggestions, to address the child’s oral health challenges. It used a decision tree with recommended responses along with natural language understanding [37]. A pediatric dentist and a community expert evaluated the content validity, theoretical basis, and contextual appropriateness. A preliminary usability study with 10 caregivers evaluated content understanding and user experience.

### Chatbot Evaluation

Chatbot evaluation should assess user patterns, user experience, conversation quality, perceived relational ability, and behavior outcomes [11,31]. This chatbot was evaluated on all aspects with the exception of oral health examinations due to COVID-19 pandemic operation limitations.

### Participants and Recruitment

From December 2021 to February 2022, a total of 66 caregivers were recruited using convenience sampling including 29 caregivers from the Early Childhood Development Center at the Faculty of Medicine, Prince of Songkla University, Hat Yai District, Songkhla Province, and 37 caregivers from the Early Childhood Development Center in Maelan District, Pattani Province, Thailand. The inclusion criteria for caregivers were as follows: (1) currently caring for children aged 6 to 36 months with at least 1 tooth; (2) their children had no disabilities or significant underlying medical conditions that could have affected their oral health; and (3) equipped with electronic devices such as computers, tablets, or mobile phones, allowing for daily internet access and a propensity to interact via Facebook Messenger. The exclusion criteria were (1) caregivers who could not speak, read, or write Thai and (2) caregivers who were opposed to the data collection.

The sample size was calculated using G*Power software (version 3.1; Heinrich Heine University Dusseldorf) to determine the mean difference in the frequency of tooth brushing per day between pre- and postintervention time points. An effect size of 0.4283, derived from unpublished data of a previous multicenter study conducted across 6 provinces in southern Thailand that evaluated the effectiveness of a chatbot in promoting tooth-brushing behaviors among 398 children aged 6 months to 2 years [41], was used. Additional parameters included a correlation coefficient of 0.5, an α level of .05, and a power of 0.8. The calculation indicated a minimum of 60 participants was required. To account for a 10% dropout rate, the target sample size was increased to 66 participants to ensure sufficient data for analysis.

Initial contact involved outreach at childcare centers and communication with caregivers via Line or phone calls to assess eligibility and interest in participation. After identifying eligible caregivers, researchers scheduled appointments to provide detailed study information. Invitation letters were distributed by the research team (MultimediaAppendices 1  2).

### Data Collection

#### Self-Reported Oral Health Behavior, Usability, and Acceptability

At baseline and 2 months later, a structured self-administered questionnaire (MultimediaAppendices 3  4) via Google Forms gathered information regarding participant characteristics and the effectiveness of chatbot use in terms of tooth-brushing behaviors and PMT-based oral health perceptions. At 2 months, usability and overall user satisfaction measures were added.

Oral hygiene behaviors were assessed using categorical questions on tooth cleaning methods, fluoride toothpaste use, and toothpaste amount.

Oral health perceptions were assessed using 14 items based on PMT including perceived severity (5 items), vulnerability (1 item), response efficacy (4 items), and self-efficacy (4 items), measured on a 5-point Likert scale.

A 5-point Likert scale, ranging from 1 (lowest) to 5 (highest), was used for all variables. The mean (SD) for each group was calculated by averaging the total items in each category. Negative items were reverse-scored before calculating the mean. The mean scores were interpreted as follows: 1.0‐1.79 were the lowest perceptions, 1.8‐2.59 were low, 2.6‐3.39 were moderate, 3.4‐4.19 were high, and 4.2‐5.0 were highest.

The usability test was designed in relation to models of Denecke et al [31] and Zhang et al [11], consisting of 15 items. This measured user experience (3 items), conversation quality (4 items), perception of relation and capacity (2 items), self-esteem (4 items), and usefulness (2 items) on a 5-point Likert scale with the same calculation and interpretation methods as shown above. In addition, the engagement was quantified by analyzing use days from chatbot logfiles.

The assessment of acceptability was conducted using 3 different methodologies. First, on day 30, participants interacted with the chatbot, responding to open-ended prompts designed to elicit both positive and negative use experiences. Second, a structured questionnaire was administered 2 months post baseline. It consisted of 1 item evaluating overall user satisfaction using a 5-point Likert scale, ranging from 1 (lowest) to 5 (highest).

Two weeks post intervention, semistructured in-depth telephone interviews were conducted. Participants (n=15) were stratified into 4 groups based on adherence levels and were randomly selected from the Hatyai and Maelan districts with equal distribution: 100% adherence (30 d, n=4), 80%‐99% adherence (24‐29 d, n=4), 50%‐79% adherence (15‐23 d, n=4), and <50% adherence (<15 d, n=3).

Participants were contacted to schedule interviews. Nonresponders were replaced through random selection until quotas were met, except in group 4, where participants were unreachable. Interviews, lasting 10‐15 minutes, covered the study’s objectives; interview methods; and explored satisfaction, dissatisfaction, feedback, and suggestions. Field notes were recorded during each interview.

#### Quality Control of Questionnaire and In-Depth Interview

The content and construct validity of the questionnaires and guiding questions for in-depth interviews were evaluated by 3 experts: a community dentist, a pediatric dentist, and a dentist from a public hospital. The usability test, adapted from Denecke et al [31] and Zhang model frameworks [11], was validated by the same 3 experts. Reliability for PMT-based perceptions was determined using Cronbach α (*α*=0.85). Face validity of the overall questionnaire was established through pilot telephone interviews with 10 caregivers. The in-depth interview guidelines were validated by 3 experts. To ensure triangulation, 2 additional independent researchers conducted content analysis. Multiple data collection methods were used to assess acceptability.

### Statistical Analysis

Descriptive statistics were presented as means (SD) for continuous variables, and frequency (percentage) for categorical variables. Within-group comparisons were used for the nominal/categorical data, and chi-square or Fisher exact tests were used. For continuous data, paired *t* tests were used. Satisfaction scores were reported as the mean (SD) on a 5-point scale. IBM SPSS Statistics for Windows (version 29.0.0.0 (241); IBM Corp) was used.

### Analysis of Qualitative Data

The responses of the participants from in-depth interviews were analyzed thematically using an inductive method guided by the procedure outlined by Jang et al [42] and Fitzpatrick et al [37]. Data from in-depth interviews and open-ended queries were analyzed using thematic analysis [43,44] and reported in terms of frequency.

### Ethical Considerations

Adhering to social distancing measures recommended during the COVID-19 pandemic, participants attended group Zoom information sessions where researchers explained the study comprehensively. To ensure privacy, attendees could use pseudonyms and disable cameras. The researcher outlined the following: (1) study purpose; (2) data handling procedures; (3) privacy measures; and (4) participant rights, including voluntary participation, and withdrawal options. Questions could be asked during the session or privately via Zoom chat. Verbal consent was obtained during the session, with the completion of the web-based questionnaire (Google Forms) serving as additional confirmation. Participants could withdraw by not completing the questionnaire. Privacy measures included guidelines against sharing others' identities or comments outside the session, and secure data storage with restricted access to research team members only. The protocol was registered to the Thai Clinical Trials Registry (TCTR20210927004) and approved by the Institutional Review Board of the Faculty of Dentistry at Prince of Songkla University (EC6407-053).

## Results

### Characteristics of Participants at the Baseline

Initially, 66 individuals were enrolled in the study, but following the 30-day postintervention period, 8 enrollees were excluded due to loss of follow-up.

The demographic and socioeconomic status of participants (n=58) were presented below (Table 1). The mean participant age was 34.5 (SD 8.6) years, and 87.9% (n=51) were mothers. The mean child age was 20.9 (SD 7.9) months. Of the participants, 55.2% (n=32) participants obtained oral hygiene advice via the web. Most web-based use was 51.7% (n=30) of the participants for between 3 and 5 hours per day, then >6 hours per day (15/58, 25.9%) and followed by 1‐2 hours per day (13/58, 22.4%). Daily internet use was 72.4% (n=42) during the week.

**Table 1.** Demographic and socioeconomic characteristics of study participants (N=58).

Demographic characteristics	Values, n (%)
**Caregiver’s education level**	
Lower than Bachelor degree	26 (44.8)
Bachelor degree or higher	32 (55.2)
**Religion**	
Buddhism	26 (44.8)
Islam	32 (55.2)
**Caregiver’s occupation**	
Stay-at-home parent	18 (31)
Civil servant	23 (39.7)
Employee	8 (13.8)
Private sector employee	4 (6.9)
Farmer	2 (3.4)
Business owner	3 (5.2)
**Family income**	
Not enough	5 (8.6)
Enough	53 (91.4)

### Oral Health Behaviors

Results from the posttest showed that tooth brushing by caregivers and the use of fluoride toothpaste and smear-sized toothpaste improved significantly (Table 2).

**Table 2.** Oral health behaviors among study participants at baseline and after 2 months from the first day of intervention (N=58). Fisher exact test significance level: *P*<.05.

Behaviors	Preintervention, n (%)	Postintervention, n (%)	*P* value
**Teeth cleaning method**			.006
Improper or no tooth brushing^a^	16 (27.6)	4 (6.9)	
Tooth brushing by caregiver	42 (72.4)	54 (93.1)	
**Frequency of tooth brushing**			<.001
<1 time/day	21 (36.2)	8 (13.8)	
≥2 times/day	37 (63.8)	50 (86.2)	
Use of Fluoride toothpaste (Yes)	31 (53.4)	49 (84.5)	.001
Smear-sized toothpaste used	34 (58.6)	48 (82.8)	.008

a Improper tooth brushing is defined as insufficient cleaning, rinsing with water only, using a cloth for cleaning, or allowing the child to brush independently.

### Perceptions of Caregivers Based on PMT

All perceptions of caregivers based on PMT showed significant improvement from high to highest perceptions, except for perceived vulnerability, which improved but without a statistically significant difference (Table 3).

**Table 3.** Perceptions of caregivers based on protection motivation theory (PMT) toward oral health behavior (total score=5). Paired *t* test significance level: *P*<.05.

	Preintervention, mean (SD)	Postintervention, mean (SD)	*P* value
**PMT parameters**			
Perceived severity	4.0 (0.9)	4.5 (0.7)	<.001
Perceived vulnerability	4.3 (1.1)	4.6 (0.9)	.56
Response-efficacy	3.9 (0.8)	4.4 (0.8)	.04
Self-efficacy	3.9 (0.9)	4.3 (0.7)	.001
Overall	4.0 (0.6)	4.5 (0.6)	<.001

### Usability and Engagement

The usability levels were highest in every category (Table 4). Of those who completed the program, 32.8% of the study participants were fully engaged in using the chatbot. The average chatbot user engagement was 24.7 days (SD 7.2), with an additional insight into weekly engagement of 5.8 days (SD 1.7).

**Table 4.** Usability on the chatbot (N=58).

Usability items (total score=5)	Values, mean (SD)
**User experiences**	4.6 (0.6)
Convenience	4.6 (0.7)
Acceptable performance and acceptance	4.6 (0.7)
**Conversational quality**	4.7 (0.6)
Understandable media	4.7 (0.6)
Reliable content	4.8 (0.7)
Appropriate content order	4.6 (0.8)
Linguistic features, naturalness, and fluency conversational quality	4.8 (0.6)
**Perception of relational and capacity**	4.7 (0.6)
Rapport perception of relation and capacity	4.6 (0.8)
Self-efficacy and perceived social support	4.8 (0.6)
Increased self-esteem	4.6 (0.6)
Usefulness of chatbot	4.8 (0.5)
Overall average score	4.7 (0.5)

### Acceptability

**Figure 4.**
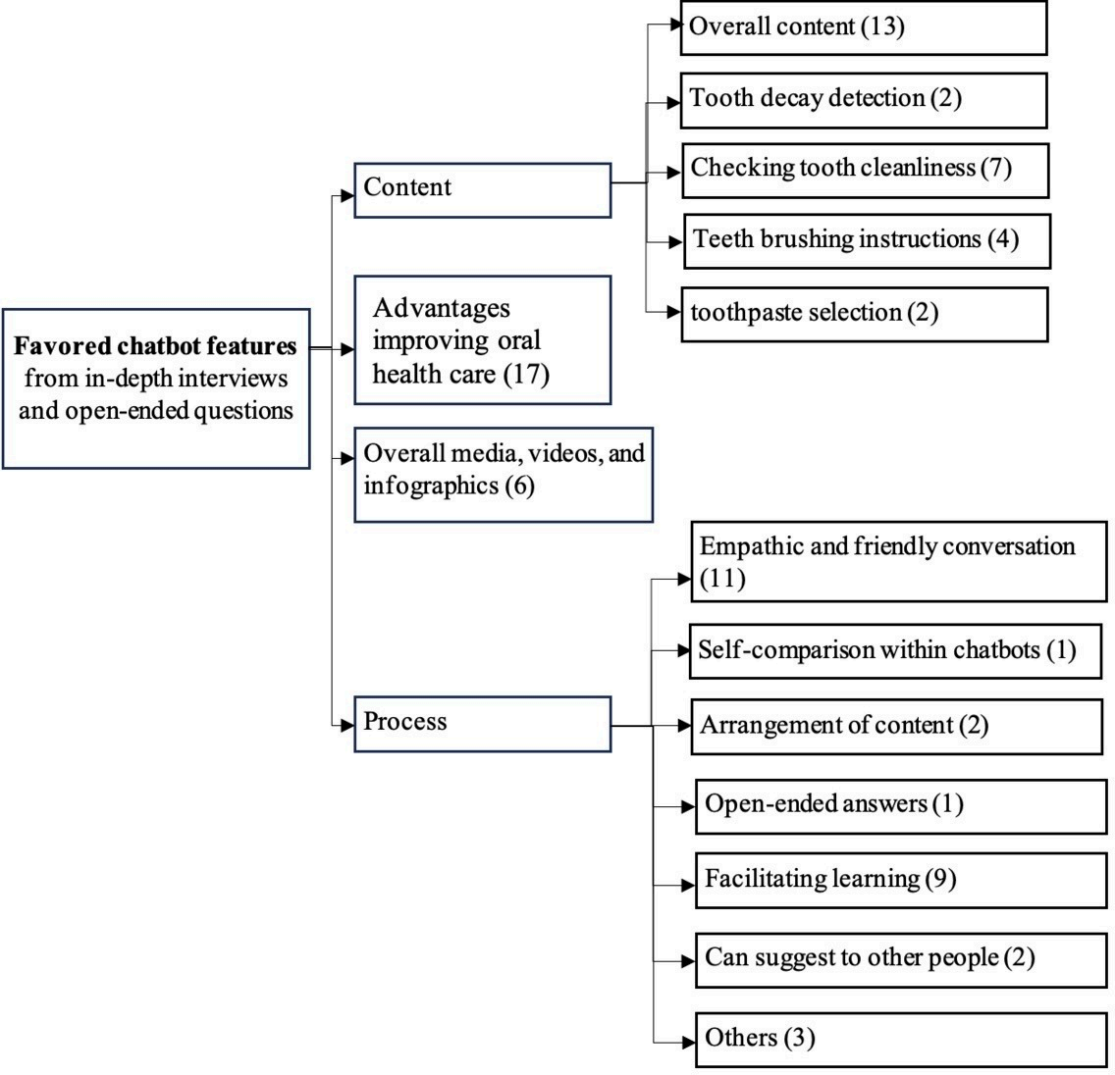
Thematic map of favored chatbot features of chatbot users’ experience, drawn from in-depth interviews and open-ended questions. (Number in bracket: number of users).

**Figure 5.**
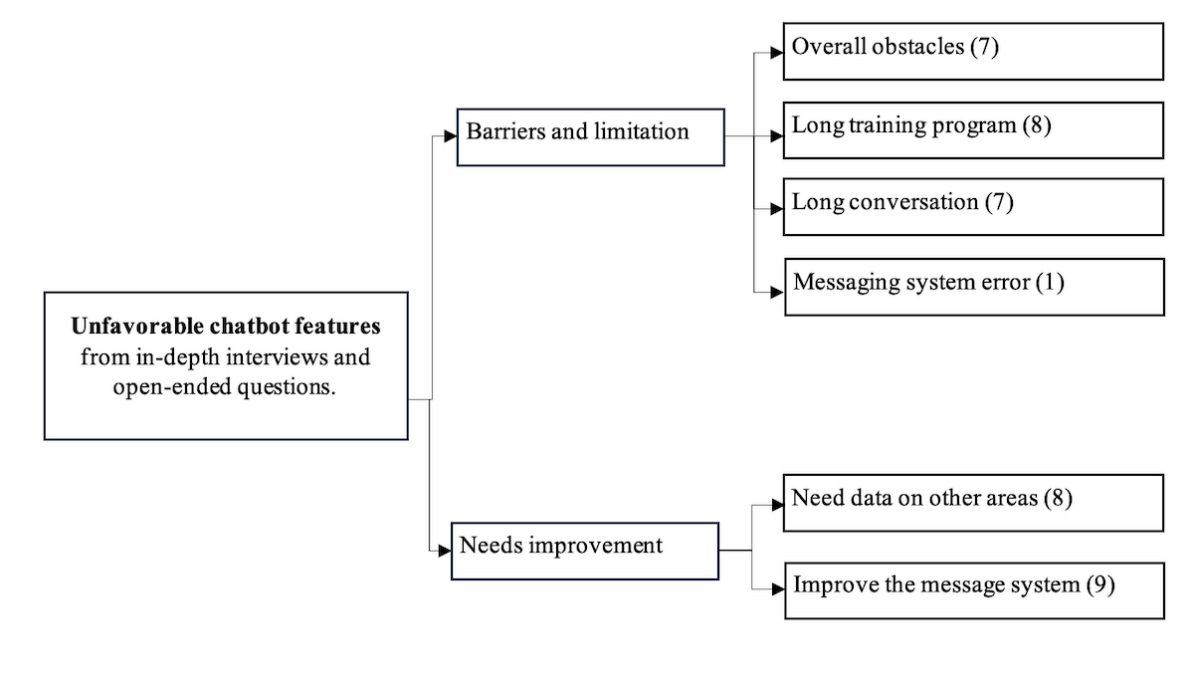
Thematic map of unfavorable chatbot features of chatbot users’ experience, drawn from in-depth interviews and open-ended questions. (Number in bracket: number of users).

In the structured questionnaire, overall satisfaction was rated 4.7 out of 5 (SD 0.6). Open-ended questions from day 30 of chatbot conversations (42 users) and in-depth interviews (15 users) revealed 4 major themes through thematic analysis: content, user learning, media, and process (Figure 4). Most users expressed positive feedback on the value of the content and the engagement methods, particularly empathetic communication and media. However, the duration of chatbot interactions and issues with the messaging system were identified as key barriers to sustained engagement (Figure 5).

## Discussion

### Principal Findings

The study indicates that using the “30-Day FunDee” chatbot significantly improved PMT-related perceptions and tooth-brushing behavior for young children. High levels of acceptability and usability were reported for the chatbot acceptability and usability were reported.

Several studies supported that knowledge and attitudes regarding tooth brushing can evolve and that alterations in tooth-brushing practices are influenced by or linked to this factor [45,46]. Caregiver-led tooth brushing for children twice daily with a rice-sized amount of fluoridated toothpaste has been proven to be a key strategy for preventing dental caries in children younger than 3 years [12]. In the study, tooth brushing by caregivers increased significantly from 72.4% to 93.1% (a significant increase of approximately 22%). The dedication of caregivers to twice-daily brushing and the use of fluoride toothpaste for children significantly grew, with an increase of approximately 22.4% (from 63.8% to 86.2%) and 31.1% (from 53.4% to 84.5%), respectively. These results go beyond those of other studies involving similar age groups, whether they used traditional oral health education with or without in-person tooth-brushing training [22,47].

Oral health professionals should provide caregivers with practical training and empowerment regarding the prevention of tooth decay in young children [15,16,48]. Within the framework of this chatbot interface, caregivers are expected to autonomously acquire the skill of proper tooth brushing through the use of educational content, encompassing videos, images, and textual information. The resultant feedback on this intervention demonstrated noteworthy effectiveness, reflecting an elevated tooth-brushing behavior and satisfaction level, quantified with a score of 4.7 out of 5. Additionally, media preferences were thoroughly explored through in-depth interviews and open-ended questions within the chatbot interface.

Meta-analyses indicate that 4 key factors within the PMT including perceived severity, perceived vulnerability, response efficacy, and self-efficacy contribute significantly to changes in health behaviors [49]. Perceived severity significantly increased, likely due to integrating PMT principles into the chatbot design and content structure. As in prior research, the perceived severity of the condition (cancer) impacted intentions to prevent disease, suggesting fear appeals influenced the perceived threat of disease [50]. In addition, the systematic review identified self-efficacy within the PMT’s coping appraisal construct as the most effective predictor and promoter of physical activity participation [51]. Our findings align with these studies, demonstrating improvements in perceived severity, response efficacy, and self-efficacy.

In this chatbot on perceived vulnerability, we requested caregivers evaluate their children’s dental plaque to determine their abilities in tooth brushing. We hypothesized that there would be a significant difference in perceived vulnerability, however this was not supported. Nevertheless, such interactions still improve oral health behaviors in children [27]. Caregivers, with a strong belief in response efficacy, are more likely to change their behavior to take care of their children [27,52]. In this study, we aimed to share tooth-brushing techniques using various media such as conversational text, infographics, both real-life and animated images, and videos. This finding is consistent with the excellent level of user satisfaction observed regarding the understandable media and reliable content displayed. Self-efficacy is a significant component that plays a critical role in influencing the improvement of health behavior [27,45,46,53].

Consistently maintaining oral health behaviors, crucial for the success of preventing caries, should be upheld over an extended period [54]. As habit formation typically takes place within a range of 18 to 254 days [19], we anticipate that the 30-day exposure to the intervention and the subsequent 60 days from the first day of intervention to the evaluation day will sufficiently ensure a lasting behavioral change.

Behavior change theories were recommended in interventions promoting change, including in-person and digital formats. These approaches emphasized goal-setting, self-monitoring, reviewing goals, addressing obstacles, providing motivation, feedback, social support, and individualized guidance and education [21,55,56]. The 30-Day FunDee chatbot study incorporated these techniques.

Integrating behavior change methods into chatbot interventions likely improved targeted behaviors and overcame limitations of conventional in-person interventions, including restricted health care professional interaction, declining motivation over time, and insufficient access to education. Thus, chatbots may elicit positive behavior changes by addressing the restrictions of traditional approaches [3].

The research involves designing a satisfaction assessment using a web-based questionnaire that was applied to evaluate the design of the AI behavior change model [11]. The evaluation focused on user experiences, conversational quality, perception of relational capacity, self-esteem enhancement, and chatbot usefulness. The results indicate the highest satisfaction across all evaluated criteria and that it was effective enough to increase tooth brushing behavior. Powerful chatbots, such as the 30-Day FunDee chatbot, are not only capable of simple conversation, but also of oral health education and self-training.

The average duration of chatbot engagement, which was 5.8 days per week in this study, is a salient feature. Due to the increased level of satisfaction, this duration is especially noteworthy; it exceeds Todaki’s 5.1 days-per-week engagement during a similar 4-week study period [42], but is inferior to Woebot’s 6.1 days-per-week engagement in a shorter 2-week study period [37]. Observations from both our own investigations and other research [42] indicate that the chatbot exhibited notable use during the initial week, followed by a decline in use from the second to the fourth week. User feedback revealed 3 key reasons: excessive workload strain, preference for shorter programs, and reliance on chatbot notifications. These findings indicate a misalignment between chatbot functionality and user needs, consistent with previous research highlighting drawbacks such as extended training programs and technical errors [37,42]. Future iterations will prioritize content condensation, enhanced user engagement, and potential platform optimization or replacement to address these challenges and improve overall chatbot efficacy.

According to the challenges of chatbot development in public health, establishing rapport and cultivating relationships with users through compassionate and personalized interactions is critical for a sustained and engaging intervention [3,4,57]. This study aligns with previous research findings, where the term “empathic and friendly conversation” emerged during in-depth interviews and open-ended questions. Similarly, studies on other chatbots corroborated these benefits [37,42,58].

The strengths of this study focus on systematically planning, developing, analyzing, and assessing the chatbot. It was meticulously designed based on behavior modification theory and principles of AI chatbot behavior change. This resulted in profound understanding, facilitating improvement, development, testing, and evaluation of interactive programs to align with the goals. This included the capacity to discuss, summarize outcomes, and conduct extensive comparative analyses with other study findings. The ultimate goal is to formulate guidelines for the next phase of chatbot development.

Our research possesses limitations due to a pre-post design, potentially introducing a maturity bias and consequently, possibly overestimating the chatbot’s effectiveness in enhancing oral health behavior. In the research methodology, a self-administered web-based questionnaire was used, raising concerns about compromised validity.

In order to improve the applicability of our results, we recommend that future research use randomized trials involving a wide range of demographic groups and conduct a more extensive evaluation of caries prevention. Furthermore, to enhance the effectiveness, user-friendliness, and acceptance of chatbots, efforts should be made to incorporate adaptive learning and AI-driven conversational methods.

### Conclusion

In summary, this research suggests that the 30-Day FunDee chatbot can be an effective, highly usable, and acceptable resource for individuals seeking oral health information and children’s caries prevention. Such a tool could be developed in the next phase of chatbots to serve as a scalable complement to conventional treatment approaches.

## Supplementary material

10.2196/62738 Multimedia Appendix 1 English invitation letter.

10.2196/62738 Multimedia Appendix 2 Thai invitation letter.

10.2196/62738 Multimedia Appendix 3 English structured self-administered questionnaire.

10.2196/62738 Multimedia Appendix 4 Thai structured self-administered questionnaire.

## Abbreviations

AI: artificial intelligence
ECC: early childhood caries
PMT: protection motivation theory
