# Supplementary material for: Development of Chatbot-Based Oral Health Care for Young Children and Evaluation of its Effectiveness, Usability, and Acceptability: Mixed Methods Study
Source: JMIR Pediatr Parent. 2025 Feb 3;8:e62738. doi: 10.2196/62738 (PMC11809939; doi:10.2196/62738)
Supplement: Multimedia Appendix 1 [file pediatrics-v8-e62738-s001.docx]

## **Invitation**

###### Research Participation Invitation

###### Study Title: Development and Effect of Chatbot 30 Days on Toothbrushing Behavior of

###### Caregivers towards 6-36 Month-Old Children.

**Dear Primary Caregiver**,

You are invited to participate in a research study conducted by Dr. Kittiwara Pupong, a post-graduate student in the Oral Health Sciences Program, Preventive Dentistry (Pediatric Dentistry), Faculty of Dentistry, Prince of Songkla University. This study is supervised by Asst. Prof. Dr. Jaranya Hunsrisakhun and Asst. Prof. Samerchit Pitpornchaikul and funded by the Faculty of Dentistry, Prince of Songkla University.

**Study Purpose**

This study aims to develop and assess the feasibility of a culturally appropriate oral health education program to enhance caregivers' knowledge, skills, and abilities in caring for the oral health of children aged 6-36 months.

**Study Procedures**

If you choose to participate, you will:

1. Use the "30-Days FunDee Chatbot" via Facebook Messenger for 30 days (3-5 minutes daily).
2. Complete online questionnaires via Google Forms before and after the intervention (approximately 10 minutes each).
3. Participate in a telephone interview at the end of the study (approximately 5-10 minutes).

The total duration of your involvement will be approximately 2 months.

**Potential Benefits**

Participants may gain improved knowledge and skills in child oral health care. Your feedback will contribute to the development of novel dental health education approaches in Thailand.

**Potential Risks**

The risks associated with this study are minimal and primarily relate to the time commitment required and potential minor discomfort during questionnaire completion or interviews.

**Confidentiality**

All data collected will be kept confidential. Results will be reported in aggregate form only, with no individual data disclosed.

**Voluntary Participation**

Your participation is entirely voluntary. You may withdraw from the study at any time without penalty or loss of benefits to which you are otherwise entitled.

**Compensation**

Upon completion of the study, you will receive a children's storybook, a F.Fan Maha Sanook set, and a children's toothbrush and toothpaste set.

**Contact Information**

For questions or concerns, please contact:
Dr. Kittiwara Phupong
Pediatric Dentistry Sub-Division, Preventive Dentistry Division
Faculty of Dentistry, Prince of Songkla University
Hat Yai District, Songkhla Province
Tel: 087-9685104
Email: kitti.pupong@gmail.com

We extend our sincere gratitude for your consideration

(Dr. Kittiwara Pupong)

Principal Investigator

**Note: - Please read the message carefully before signing the consent document to participate in the project.**
