# Supplementary material for: Development of Chatbot-Based Oral Health Care for Young Children and Evaluation of its Effectiveness, Usability, and Acceptability: Mixed Methods Study
Source: JMIR Pediatr Parent. 2025 Feb 3;8:e62738. doi: 10.2196/62738 (PMC11809939; doi:10.2196/62738)
Supplement: Multimedia Appendix 2 [file pediatrics-v8-e62738-s002.docx]

## **ใบเชิญชวน**

**ขอเชิญเข้าร่วมโครงการวิจัย เรื่อง** **โปรแกรมโต้ตอบอัตโนมัติ 30 วันฟันดี ต่อการดูแลทำความสะอาดช่องปากของผู้ดูแลเด็กอายุ 6-36 เดือน: การพัฒนา และความเป็นไปได้ของการใช้งาน**

###### เรียน ท่านผู้ดูแลหลักที่นับถือ

ข้าพเจ้า ทพญ.กิตติวรา ภู่พงษ์ นักศึกษาหลักสูตรนักศึกษาหลังปริญญามหาบัณฑิต สาขาวิทยาศาสตร์สุขภาพช่องปาก สาขาวิชาทันตกรรมป้องกัน (ทันตกรรมสำหรับเด็ก) คณะทันตแพทยศาสตร์ มหาวิทยาลัยสงขลานครินทร์ (ภายใต้การดูแลของอาจารย์ที่ปรึกษา คือ ผศ.ดร.ทพญ.จรัญญา หุ่นศรีสกุล และ ผศ.ทพญ.เสมอจิต พิธพรชัยกุล) ได้รับทุนวิจัยจาก คณะทันตแพทยศาสตร์ มหาวิทยาลัยสงขลานครินทร์ ใคร่ขอเล่าถึงโครงการวิจัยที่กำลังทำอยู่ และขอเชิญชวนท่านเข้าร่วมโครงการนี้

โครงการวิจัยนี้มีเป้าหมาย เพื่อพัฒนาแนวทางการให้ทันตสุขศึกษาที่เหมาะสมกับในพื้นที่ ซึ่งจะมีประโยชน์ให้ตัวของผู้เข้าร่วมได้มีโอกาสในการได้เพิ่มทักษะ และความรู้ความสามารถในการดูแลสุขภาพช่องปากของบุตรหลานที่เกี่ยวข้องในงานวิจัยได้ และเป็นการส่งเสริมให้เด็กได้รับโอกาสในการมีช่องปากที่ดีต่อไป โดยท่านจะได้ใช้งานโปรแกรมโต้ตอบอัตโนมัติ 30 วันฟันดี (Chatbot 30 วันฟันดี) ซึ่งเป็นการพูดคุยอย่างเป็นกันเองผ่าน facebook messenger มีการเล่นเกม สอดแทรกเนื้อหาที่น่าสนใจผ่านอินโฟกราฟฟิก แอนนิเมชั่น และวิดีทัศน์ พร้อมให้กำลังใจต่อเนื่องทุกวัน เป็นเวลา 30 วัน

ทีมวิจัยจะเก็บข้อมูล ก่อน และหลังการใช้งาน เพื่อเปรียบเทียบการแปรงฟันในผู้ดูแลเด็กอายุ 6-36 เดือน ผ่านแบบสอบถาม และสัมภาษณ์ทางโทรศัพท์ ซึ่งประกอบด้วยเรื่อง ความรู้ ทัศนคติ และการปฏิบัติในการแปรงฟัน รวมถึงความพึงพอใจในการใช้งาน Chatbot 30 วันฟันดี

หลังจากที่ท่านยินยอมโดยวาจาเข้าร่วมโครงการ ท่านจะได้รับการลงทะเบียน เพื่อรับความรู้ผ่าน Chatbot 30 วัน วันละ 3-5 นาที รวมถึงตัวท่านเองจะทำแบบสอบถาม Online ผ่าน Google Forms ใช้เวลาประมาณ 10 นาที ช่วงก่อนวิจัย และสิ้นสุดการวิจัย โดยในระยะสิ้นสุดการวิจัย จะมีการเก็บข้อมูลผ่านแบบสอบถาม Online และสัมภาษณ์ความพึงพอใจผ่านโทรศัพท์ ใช้เวลาประมาณ 5-10 นาที การเก็บข้อมูลห่างจากครั้งแรก (ก่อนวิจัย) ประมาณ 2 เดือน

หากท่านตัดสินใจเข้าร่วมโครงการวิจัยในครั้งนี้ นั่นหมายถึงท่านจะได้ทำแบบสอบถาม Online ผ่าน Google Forms จำนวน 2 ครั้ง และได้รับการสัมภาษณ์ทางโทรศัพท์ 1 ครั้ง โดยท่านจะได้รับของที่ระลึกเป็นนิทานทะลุมิติ ชุด
ฟ.ฟัน มหาสนุก ชุดแปรงสีฟัน และยาสีฟันสำหรับเด็กในครั้งสุดท้าย

งานวิจัยนี้มีโอกาสที่จะเกิดความเสี่ยง และอันตรายขณะเก็บข้อมูลมีน้อยมาก คือ การตอบแบบสอบถาม และการสัมภาษณ์ผู้ดูแลเด็ก ซึ่งอาจทำให้เสียเวลา เกิดความไม่สะดวก หรือกังวลใจบ้างเล็กน้อย ดังนั้นเพื่อเป็นการป้องกันการละเมิดจริยธรรม ผู้วิจัยจะชี้แจงให้ท่านทราบก่อนล่วงหน้า

การเข้าร่วมโครงการ และการให้ข้อคิดเห็นของท่านจะเป็นแนวทางการพัฒนาการให้ทันตสุขศึกษาแนวใหม่ของสังคมไทยในยุควิถีใหม่ (New normal) ต่อไป

ข้อมูลทั้งหมดที่ได้จากการวิจัยจะเก็บเป็นความลับ จะไม่เปิดเผยข้อมูลหรือผลการวิจัยเป็นรายบุคคลต่อสาธารณชน จะเปิดเผยในรูปแบบที่เป็นการประมวลผล และสรุปผลการวิจัย หรือเปิดเผยข้อมูลต่อผู้ที่มีหน้าที่เกี่ยวข้องกับการสนับสนุนและกำกับดูแลการวิจัยเท่านั้น

ไม่ว่าท่านจะเข้าร่วมในโครงการวิจัยนี้หรือไม่ ท่านจะยังคงได้รับการรักษาที่ดีเช่นเดียวกับผู้ป่วยคนอื่นๆ และถ้าท่านต้องการที่จะถอนตัวออกจากการศึกษานี้เมื่อใด ท่านก็สามารถกระทำได้อย่างอิสระ

มีต่อหน้าที่ 2

หากท่านมีคำถามใด ๆ ก่อนที่จะตัดสินใจเข้าร่วมโครงการนี้ โปรดซักถามคณะผู้วิจัยได้อย่างเต็มที่ โดยสามารถติดต่อได้ที่ ทพญ.กิตติวรา ภู่พงษ์ อนุสาขาวิชาทันตกรรมสำหรับเด็ก สาขาวิชาทันตกรรมป้องกัน คณะทันตแพทยศาสตร์มหาวิทยาลัยสงขลานครินทร์ อำเภอหาดใหญ่ จังหวัดสงขลา โทร 087-9685104 หรือ e-mail : [kitti.pupong@gmail.com](mailto:kitti.pupong@gmail.com)

ขอขอบคุณเป็นอย่างสูง

(ทพญ.กิตติวรา ภู่พงษ์)

หัวหน้าโครงการ

**หมายเหตุ :- กรุณาอ่านข้อความให้เข้าใจก่อนเซ็นชื่อเอกสารให้ความยินยอมเข้าร่วมโครงการ**
