# Supplementary material for: Development of Chatbot-Based Oral Health Care for Young Children and Evaluation of its Effectiveness, Usability, and Acceptability: Mixed Methods Study
Source: JMIR Pediatr Parent. 2025 Feb 3;8:e62738. doi: 10.2196/62738 (PMC11809939; doi:10.2196/62738)
Supplement: Multimedia Appendix 3 [file pediatrics-v8-e62738-s003.docx]

**Interview form: General information, behavior, knowledge and attitude towards oral health care.**

Day/Month/Year _ _ / _ _ / _ _

**Section 1 General information**

**General information for caregivers**

**Your name is................... Please use the pronoun to call you......................**

**My child's nickname is........................, age..................years.**

| 1. You... ( name )…. what's your relationship with the child?  \| 1 □mother \| 2□ father \| 3□ grandmother, grandfather, \| \| --- \| --- \| --- \| \| 4□ Uncle, aunt \| 5□ Other, please specify \|  \| |
| --- | --- | --- | --- | --- | --- | --- |
| 1. You... ( use a pronoun )…. How old are you ( if you can't remember) ? Please ask the year of birth. )   Age of caregiver...........years  Or born in the year B.E. ................... |
| 1. What the grade did you graduate from?  \| 1□ Primary education \| 2□ Lower secondary school \| 3□ High school / Vocational Certificate \| \| --- \| --- \| --- \| \| 4□ Associate Degree / Advanced Vocational Certificate \| 5□ Bachelor's degree \| 6□ Higher than bachelor's degree \| \| 7□ Other, please specify................. \| \| \| |
| 1. When comparing your family income with your expenses, is it (providing the choices)?  \| 1□ Not enough \| 2□ Enough, but there is nothing left to save. \| 3□ Enough, and there is some left over \| \| --- \| --- \| --- \| |
| 1. What is your occupation?  \| 1□ Homemaker or unemployed \| 2□ Government service / State enterprise \| 3□ General employee \| \| --- \| --- \| --- \| \| 4□ Company employee \| 5□ Agriculturalist \| 6□ Small Business / Business Owner \| \| 7□ Other, please specify................. \| \| \| |
| 1. What religion do you practice?  \| 1□ Islam \| 2□ Buddhism \| 3□ Christianity \| \| --- \| --- \| --- \| \| 4□ Others, please specify........................................................ \| \| \| |
| 1. **This child** is the ……………………………….. **Child. (Birth ranking)** |
| 1. **Total** number of children in household (including those in this study) …………….. people |
| 1. Number of people the caregiver must care for (all ages included) But not including the total number of children in section 8 ) such as children, the elderly, bedridden patients, or people with chronic diseases who cannot take care of themselves …………………………. people |
| 1. Have you ever received any advice about brushing teeth or taking care of oral health in young children?  \| 1. □ I have. \| 2. □ I have never **(Skip to question 12)** \|  \| \| --- \| --- \| --- \| |
| 1. Have you ever received advice on how to brush teeth or care for oral health in young children? If so, from what sources? **(You can answer more than one)**  \| 1. □**Public health officials**   - 1□ Dental personnel - 1□ Nurse - 1□ Subdistrict Health Promoting Hospital Staff - 1□ Village Health Volunteers - 1□ other ....... \| 2□ **On the internet** , such as   - 2□ Facebook​​ - 2□ Line​ - 2□ Website​ - 2□ YouTube​​​ - 2□ other ......... \| \| --- \| --- \| \| 3□ Other channels   - 3□ A Poster received from......... - 3□ A brochure received from......... - 3□ Radio - 3□ Television \| 4□ Other channels not listed in the options, please specify............. \| |
| 1. Nowadays, do you...access the Internet via your mobile phone, computer or other devices? **Or not?** If yes, **how many hours per day** on average?   (If you don't use the internet at all, record it as 0 hours and **skip to step 14.** )  Use the Internet for.........hours per day. |
| 1. ( Continued from question 12) If accessed, **how many days** on average **per week ?**   Use the internet.......days per week  **2. Oral health care behavior** |

| 1. In the past week has the child received or had an oral cleaning (if so , how often ) ?  \| 1. □Not cleaned yet ( skip to **Item 22)** ) \| \| --- \| \| 2. □Use a wet cloth to clean their mouth ( skip to **Item 22** ) \| \| 3 □Children brush their teeth by themselves ( skip to **Item 22** ) \| \| 4. □You... brush the child's teeth or the child brushes by himself and you... brush again. \| |
| --- | --- | --- | --- | --- |
| 1. In the past week, **how many days / week have you brushed your child's teeth?**   Brushed child’s teeth............days / week |
| 1. In the past week, **how many times per day have you brushed your child's teeth?**   Brush your teeth...........times / day |
| 1. In an average day, when do you usually brush your child's teeth? **You can answer more than one question.**  \| 1 □morning \| 2 □noon \| 3 □night \| \| --- \| --- \| --- \| \| 4. □Before bed \| 5□ Other, please specify................. \|  \| |
| 1. After the last brushing of the day, do you still give your child milk, snacks, or other food besides water before bed?  \| 1. □Don't give \| 2□ Give................ ( Please specify ) \| \| --- \| --- \| |
| 1. Do you brush your child's teeth using toothpaste?  \| 1. □Do not use toothpaste ( **skip to step 22)** at all ) \| \| --- \| \| 2. □Use toothpaste . \| |
| 1. Have you ever looked at the side of the box to see if the toothpaste you use to brush your child's teeth contains fluoride?  \| 1. □Contains fluoride \| 2. □No fluoride \| 3. □Can't remember /not sure \| \| --- \| --- \| --- \| |
| 1. What is the amount of toothpaste you use on this child ?  \| 1. □Apply a thin layer to wet the brush / rice grain sized.  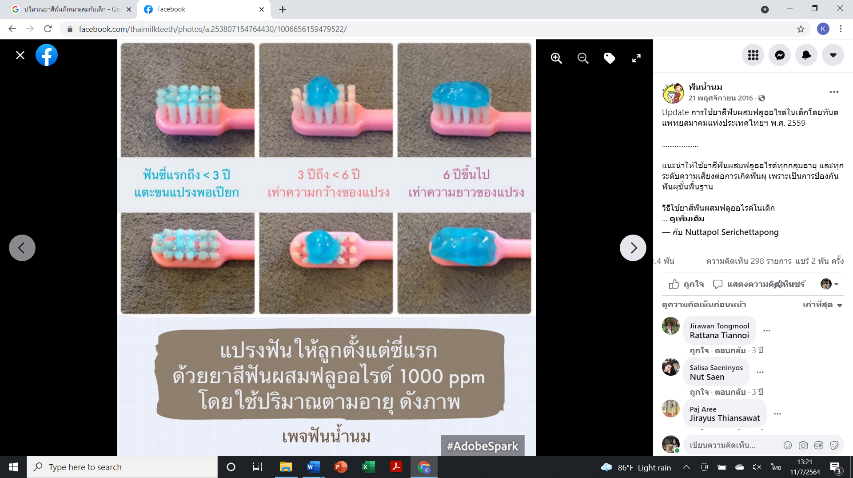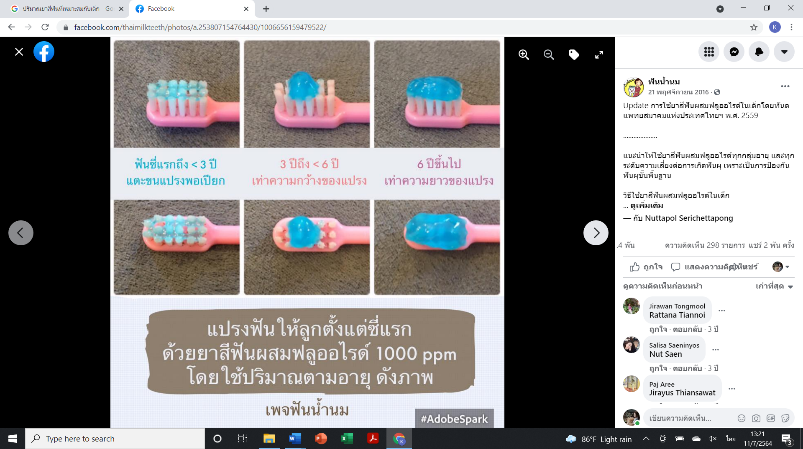 \| 2 □brush width  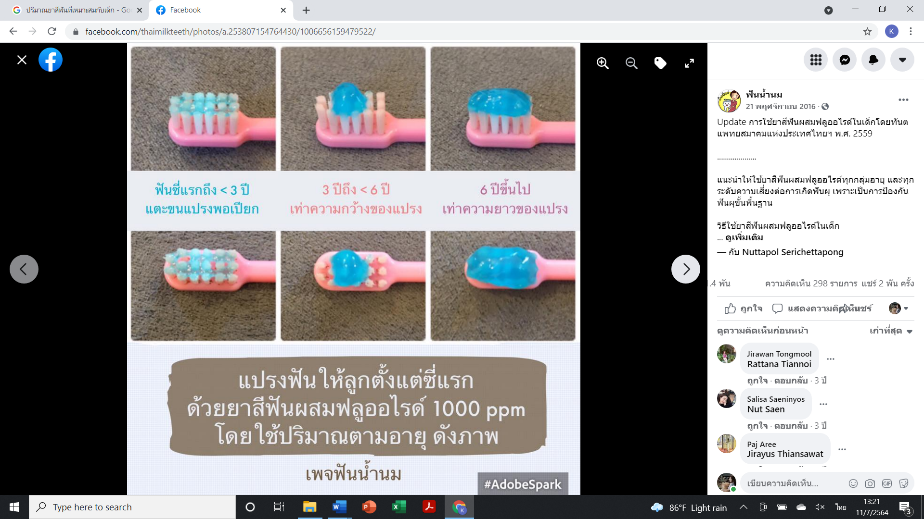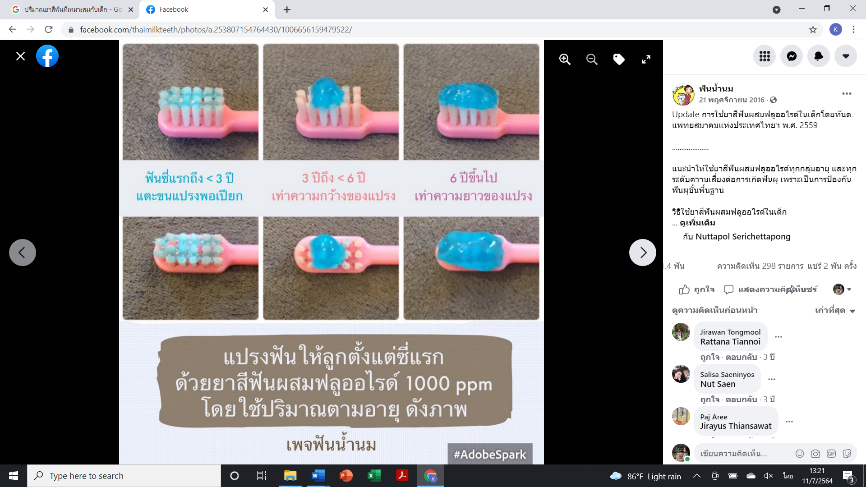 \| 3 □brush length  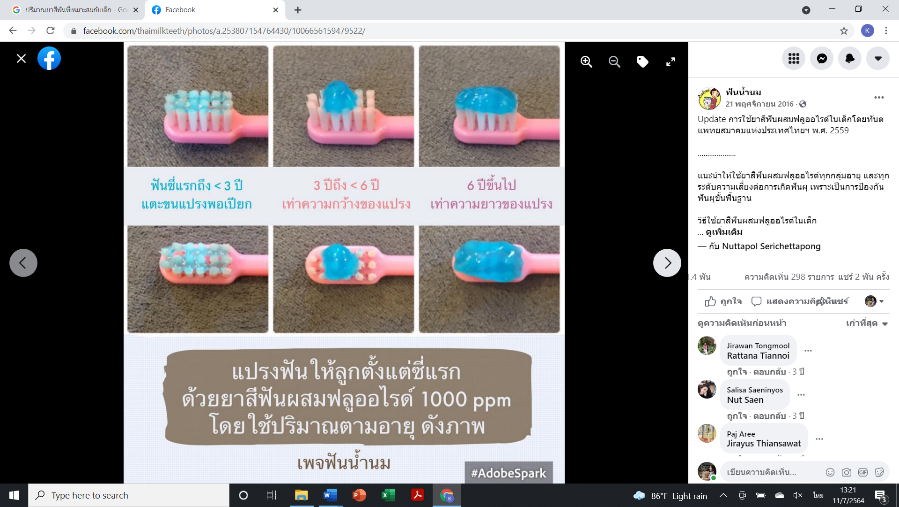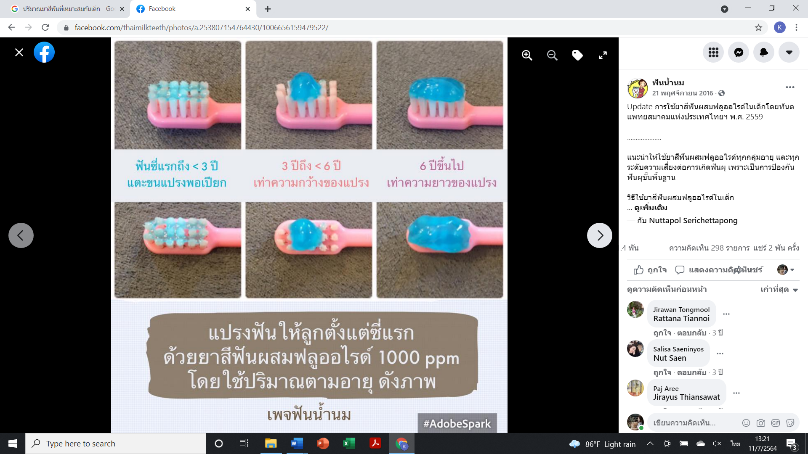 \| \| --- \| --- \| --- \| |

**3. Oral health care attitudes**

Do you...agree with the following statement?

If you agree, answer agree.

If you totally agree, answer totally agree.

If you disagree, answer disagree.

If you strongly disagree, answer strongly disagree.

If you are not sure, answer not sure.

| **number** | **question** | **feeling** | | | | |
| --- | --- | --- | --- | --- | --- | --- |
|  |  | **1**  **strongly disagree.** | **2**  **Disagree** | **3**  **unsure** | **4**  **agree** | **5**  **totally agree.** |
|  | It is common for children to have cavities in their baby teeth. |  |  |  |  |  |
|  | Tooth decay is a disease of the teeth only. It does not affect other parts of the body. |  |  |  |  |  |
|  | It's okay if your baby teeth decay. It won't take long for your permanent teeth to replace them. There are no negative effects. |  |  |  |  |  |
|  | Even though children have many cavities, they can still be happy (happy in a childlike way). |  |  |  |  |  |
|  | If a child has a toothache due to tooth decay, the child will be more cranky and difficult to raise. |  |  |  |  |  |
|  | Your child... already has a clean mouth. You... don't have to brush your child's teeth. |  |  |  |  |  |
|  | If the child only drinks milk, after drinking milk (including breast milk, formula milk, and other milk), there is no need to brush the teeth. |  |  |  |  |  |
|  | Putting your baby to sleep with milk will help them fall asleep easily. |  |  |  |  |  |
|  | If you brush your child's teeth every day, you will prevent tooth decay. |  |  |  |  |  |
|  | Children who have 1-2 teeth do not need to brush their teeth yet. |  |  |  |  |  |
|  | Brushing your child's teeth from the time their first tooth appears will make brushing their teeth easier when they grow up. |  |  |  |  |  |
|  | If one day you... don't have time or your child cries a lot, you can stop brushing your teeth for now. |  |  |  |  |  |
|  | If you... brush your child's teeth and their gums bleed, you...stop brushing immediately because your child will be in pain. |  |  |  |  |  |
|  | Brushing teeth for young children is more difficult than you can... do. |  |  |  |  |  |
|  | You... can brush your child's teeth even if the child cries and other people tell you to stop brushing. |  |  |  |  |  |
|  | You... can brush your child's teeth even if he cries a lot and refuses to be brushed. |  |  |  |  |  |
|  | You... can brush your child's teeth by following the instructions in the video clip |  |  |  |  |  |
|  | You... can't tell if your teeth are clean after brushing because the plaque and the teeth are the same white color and you don't know how to check the cleanliness. |  |  |  |  |  |
|  | You... are determined to brush your child's teeth with all your heart. |  |  |  |  |  |
|  | You... can give advice to others on how to brush your child's teeth. |  |  |  |  |  |

**4. Knowledge of oral health care**

Do you think the following statements are correct? Select **Yes** or **No.** If you don't know, select I **don't know.**

| **Point no.** | **question** | **answer** | | |
| --- | --- | --- | --- | --- |
|  |  | **yes**  **(1)** | **no**  **(2)** | **I don't know.**  **(3)** |
|  | If baby teeth are severely decayed, it will result in black teeth and an ugly smile. It does not have any other effects on the child. |  |  |  |
|  | Children should have their first tooth brushing when their chewing molars have erupted. |  |  |  |
|  | Children under 2 years old must brush their teeth every day. |  |  |  |
|  | Children under 2 years old should brush their teeth at least once a day. |  |  |  |
|  | When choosing a toothbrush for children, choose a hard-bristled one to remove all plaque. |  |  |  |
|  | Toothbrushes used for young children can be of the same size as for older children. |  |  |  |
|  | The right way to brush teeth for young children is to brush up and down. |  |  |  |
|  | If you are the one brushing your child's teeth, the best way to see their mouth is to lie down. |  |  |  |
|  | When you brush your child's teeth, parting the cheeks will make the teeth you want to brush more visible. |  |  |  |
|  | In young children, it is not necessary to wipe off the foam every time you brush your teeth. |  |  |  |
|  | Children can use adult toothpaste that contains fluoride without any health hazards (except for the fact that children's mouths will be irritated). |  |  |  |
|  | If your child still can't rinse, you should choose a toothpaste without fluoride. |  |  |  |
|  | If your child cries or struggles while brushing their teeth, you should not try to keep them still, as this will make them more resistant. |  |  |  |
|  | The most important thing in positioning teeth brushing in children is that the child's head must be still and the person brushing must have a clear view of the teeth to be brushed. |  |  |  |
|  | Correct tooth brushing posture will not cause pain to the child. |  |  |  |

**5. Communication satisfaction assessment form**

**Let you... choose the one that matches your idea... only 1.**

| 1. The length of daily conversations is appropriate.  \| 1□ Too long \| 2 □Suitable \| 3 □Too short. \| \| --- \| --- \| --- \| |
| --- | --- | --- | --- |
| 1. The number of days to send messages (30 days) is too much, too little, or is it appropriate?  \| 1 □Too much \| 2 □Suitable \| 3 □Too little \| \| --- \| --- \| --- \| |

**How satisfied are you with the 30 -Day Fun Dee Chatbot on the following issues? On a 5- point scale
, 5 means most satisfied, 4 means very satisfied, 3 means moderately satisfied, 2 means slightly satisfied, and 1 means least satisfied.**

| **Point no.** | **question** | **Satisfaction** | | | | |
| --- | --- | --- | --- | --- | --- | --- |
|  |  | **1**  Least Satisfied | **2**  slightly satisfied | **3**  Moderately Satisfied | **4**  Very satisfied | **5**  Most Satisfied |
|  | Easy to use or play |  |  |  |  |  |
|  | Media such as text, video, and images are easy to understand. |  |  |  |  |  |
|  | The information is reliable. |  |  |  |  |  |
|  | Speaks in a friendly, easy-to-understand language. |  |  |  |  |  |
|  | When you play with Chatbot, it feels like you're actually talking to a doctor. |  |  |  |  |  |
|  | There is encouragement to take care of children. |  |  |  |  |  |
|  | The order of the content is appropriate, making it easy to understand. |  |  |  |  |  |
|  | Chatbot response message is not too long. |  |  |  |  |  |
|  | The system sends and receives messages continuously and consistently every day for 30 days. |  |  |  |  |  |
|  | Chatbot makes you want to brush your child's teeth. |  |  |  |  |  |
|  | You... can brush your child's teeth by viewing the infographic in the Chatbot. |  |  |  |  |  |
|  | Chatbots give you the confidence to brush your child's teeth. |  |  |  |  |  |
|  | The information received from the Chatbot is useful. |  |  |  |  |  |
|  | The information from Chatbot can be applied in your daily life. |  |  |  |  |  |
|  | Overall satisfaction |  |  |  |  |  |

| **Open-ended questions** |
| --- |
| 1. Any other feelings **of satisfaction**? Please tell me.  - content   .........................................................................................................................  .................................................. .................................................. .....................   - Please comment on Chatbot design, structure or system, such as ease of use, sending messages at the same time every day, natural conversation, recording, follow-up, or attractive design.   .................................................. .................................................. .....................  .................................................. .................................................. ..................... |
| 1. Do you... have any feelings of **dissatisfaction** with Chatbot or would like to see improvements in any areas?  - content   .................................................. .................................................. ....................  .................................................. .................................................. .....................   - Please comment on Chatbot design, structure or system, such as difficult to access, missing messages, not sent every day, unnatural conversation, or unattractive design.   ........................................................................................................................  ........................................................................................................................   - Things to improve in the 30-day Fun Dee Chatbot ................................................................................................................................   .................................................................................................................................... |
| 1. Reasons to stop using 30-day Fun Dee Chatbot   ....................................................................................................................................  ..................................................................................................................... |
