# Supplementary material for: Development of Chatbot-Based Oral Health Care for Young Children and Evaluation of its Effectiveness, Usability, and Acceptability: Mixed Methods Study
Source: JMIR Pediatr Parent. 2025 Feb 3;8:e62738. doi: 10.2196/62738 (PMC11809939; doi:10.2196/62738)
Supplement: Multimedia Appendix 4 [file pediatrics-v8-e62738-s004.docx]

**แบบสัมภาษณ์ ข้อมูลทั่วไป พฤติกรรม ความรู้ และทัศนคติในการดูแลสุขภาพช่องปาก**

วัน/เดือน/ปี _ _ / _ _ / _ _

**หมวดที่ 1 ข้อมูลทั่วไป**

**ข้อมูลทั่วไปของผู้ดูแล**

**คุณชื่อ..................ให้ใช้สรรพนามเรียกแทนว่า......................**

**ลูกชื่อเล่นว่า........................อายุ..................ปี**

| 1. คุณ...(ชื่อ)….เป็นอะไรกับเด็ก  \| 1 🞏 แม่ \| 2 🞏 พ่อ \| 3 🞏 ย่า ยาย ปู่ ตา \| \| --- \| --- \| --- \| \| 4 🞏 ลุง ป้า น้า อา \| 5 🞏 อื่นๆ โปรดระบุ \|  \| |
| --- | --- | --- | --- | --- | --- | --- |
| 1. คุณ...(ใช้สรรพนาม)….อายุเท่าไหร่ (ถ้าจำไม่ได้ ขอถามปี พ.ศ.ที่เกิดหน่อยค่ะ)   อายุผู้ดูแล...........ปี  หรือ เกิดปี พ.ศ. ................... |
| 1. คุณ...เรียนจบชั้นไหน  \| 1 🞏 ประถมศึกษา \| 2 🞏 มัธยมศึกษาตอนต้น \| 3 🞏 มัธยมศึกษาตอนปลาย/ ปวช. \| \| --- \| --- \| --- \| \| 4 🞏 อนุปริญญา/ ปวส. \| 5 🞏 ปริญญาตรี \| 6 🞏 สูงกว่าปริญญาตรี \| \| 7 🞏 อื่นๆ โปรดระบุ................. \| \| \| |
| 1. รายได้ในครอบครัว เมื่อเทียบกับรายจ่ายเป็นอย่างไรบ้าง  \| 1 🞏 ไม่เพียงพอ \| 2 🞏 เพียงพอ แต่ไม่เหลือเก็บ \| 3 🞏 เพียงพอ และมีเหลือเก็บ \| \| --- \| --- \| --- \| |
| 1. คุณ....ทำงานอะไร  \| 1 🞏 แม่บ้าน/ พ่อบ้าน หรือว่างงาน \| 2 🞏 รับราชการ/ รัฐวิสาหกิจ \| 3 🞏 รับจ้างทั่วไป \| \| --- \| --- \| --- \| \| 4 🞏 พนักงานบริษัท \| 5 🞏 เกษตรกร \| 6 🞏 ค้าขาย/เจ้าของธุรกิจ \| \| 7 🞏 อื่นๆ โปรดระบุ................. \| \| \| |
| 1. คุณ...นับถือศาสนาอะไร  \| 1 🞏 อิสลาม \| 2 🞏 พุทธ \| 3 🞏 คริสต์ \| \| --- \| --- \| --- \| \| 4 🞏 อื่นๆ โปรดระบุ........................................................ \| \| \| |
| 1. **บุตรคนนี้**เป็นบุตร**คนที่**……………………………….. |
| 1. จำนวน**บุตรทั้งหมด** (รวมคนในการศึกษานี้) …………….. คน |
| 1. จำนวนคนที่ผู้ดูแลต้องดูแล (รวมทุกอายุ แต่ไม่รวมจำนวนบุตรทั้งหมดในข้อ 8) เช่น เด็ก ผู้สูงอายุ ผู้ป่วยติดเตียง หรือผู้ที่มีโรคประจำตัวที่ไม่สามารถดูแลตนเองได้ …………………………. คน |
| 1. คุณ...เคยได้รับคำแนะนำ การสอนการแปรงฟัน หรือการดูแลสุขภาพช่องปากในเด็กเล็ก หรือไม่  \| 1 🞏 เคย \| 2 🞏 ไม่เคย **(ข้ามไปข้อ 12)** \|  \| \| --- \| --- \| --- \| |
| 1. คุณ...เคยได้รับคำแนะนำเกี่ยวกับวิธีการแปรงฟัน หรือการดูแลสุขภาพช่องปากในเด็กเล็ก จากแหล่งใดบ้าง  **(ตอบได้มากกว่า 1 ข้อ)**  \| 1 🞏 ทางเจ้าหน้าที่สาธารณสุข   - 1 🞏 ทางทันตบุคลากร - 1 🞏 พยาบาล - 1 🞏 เจ้าหน้าที่ รพ.สต. - 1 🞏 อสม. - 1 🞏 อื่นๆ....... \| 2 🞏 **ทางอินเตอร์เน็ต** เช่น   - 2 🞏 เฟสบุ๊ค (Facebook) - 2 🞏 ไลน์ (Line) - 2 🞏 เว็บไซด์ (Website) - 2 🞏 ยูทูป (YouTube) - 2 🞏 อื่นๆ ......... \| \| --- \| --- \| \| 3 🞏 ช่องทางอื่นๆ   - 3 🞏 โปสเตอร์ ได้รับจาก......... - 3 🞏 แผ่นพับ ได้รับจาก......... - 3 🞏 วิทยุ - 3 🞏 โทรทัศน์ \| 4 🞏 ช่องทางอื่นๆ ที่ไม่มีในตัวเลือก โปรดระบุ............. \| |
| 1. ปัจจุบัน คุณ...ใช้อินเตอร์เน็ต ผ่านโทรศัพท์มือถือ คอมพิวเตอร์ หรืออุปกรณ์อื่นๆ **บ้างหรือไม่** ถ้าใช้ ใช้เฉลี่ยเป็นเวลา**กี่ชั่วโมง ใน 1 วัน**   (ถ้าไม่ใช้อินเตอร์เน็ตเลย บันทึกเป็น 0 ชั่วโมง และ**ข้ามไปข้อ14**)  ใช้อินเตอร์เน็ตวันละ.........ชั่วโมง |
| 1. (ต่อจากข้อ 12) ถ้าใช้ ใช้เฉลี่ยเป็นเวลา**กี่วัน ใน 1 สัปดาห์**   ใช้อินเตอร์เน็ตสัปดาห์ละ.......วัน  **2. พฤติกรรมการดูแลสุขภาพช่องปาก** |

| 1. ใน 1 สัปดาห์ที่ผ่านมา เด็กได้รับหรือมีการทำความสะอาดช่องปาก บ้างหรือไม่ (ถ้ามี ทำด้วยวิธีใดบ่อยที่สุด)  \| 1 🞏 ยังไม่ทำความสะอาด (ข้ามไปที่ **ข้อ22)**) \| \| --- \| \| 2 🞏 ใช้ผ้าชุบน้ำเช็ดทำความสะอาดช่องปาก (ข้ามไปที่ **ข้อ22**) \| \| 3 🞏 เด็กแปรงฟันด้วยตัวเอง (ข้ามไปที่ **ข้อ22**) \| \| 4 🞏 คุณ...แปรงฟันให้เด็ก หรือเด็กแปรงด้วยตนเองและคุณ...แปรงซ้ำ \| |
| --- | --- | --- | --- | --- |
| 1. ใน 1 สัปดาห์ที่ผ่านมา คุณ...แปรงฟันให้เด็ก**กี่วัน/สัปดาห์**   แปรงฟัน............วัน/สัปดาห์ |
| 1. ใน 1 สัปดาห์ที่ผ่านมา คุณ...แปรงฟันให้เด็ก**กี่ครั้งต่อวัน**   แปรงฟัน...........ครั้ง/วัน |
| 1. ในแต่ละวัน โดยส่วนใหญ่ คุณ...แปรงฟันให้เด็กตอนไหนบ้าง **ตอบได้มากกว่า 1 ข้อ**  \| 1 🞏 เช้า \| 2 🞏 เที่ยง \| 3 🞏 เย็น \| \| --- \| --- \| --- \| \| 4 🞏 ก่อนนอน \| 5 🞏 อื่นๆ โปรดระบุ................. \|  \| |
| 1. หลังการแปรงฟันครั้งสุดท้ายของวัน คุณ...ยังให้เด็กกินนม ขนม หรืออาหารอื่นๆ นอกจากน้ำเปล่า ก่อนนอนหรือไม่  \| 1 🞏 ไม่กิน \| 2 🞏 กิน...............................(โปรดระบุ) \| \| --- \| --- \| |
| 1. คุณ...แปรงฟันให้เด็ก โดยใช้ยาสีฟันหรือไม่  \| 1 🞏 ไม่ใช้ยาสีฟัน (**ข้ามไปทำข้อ 22** เลย) \| \| --- \| \| 2 🞏 ใช้ยาสีฟัน \| |
| 1. ได้เคยดูข้างกล่องมั้ยคะ ว่ายาสีฟันที่คุณ...ใช้แปรงฟันให้เด็กมีฟลูออไรด์หรือไม่  \| 1 🞏 มีฟลูออไรด์ \| 2 🞏 ไม่มีฟลูออไรด์ \| 3 🞏 จำไม่ได้ /ไม่แน่ใจ \| \| --- \| --- \| --- \| |
| 1. ปริมาณยาสีฟันที่คุณ...ใช้กับเด็ก (คนนี้) คือปริมาณเท่าใด  \| 1 🞏 แตะบางๆ พอเปียกแปรง/เม็ดข้าว  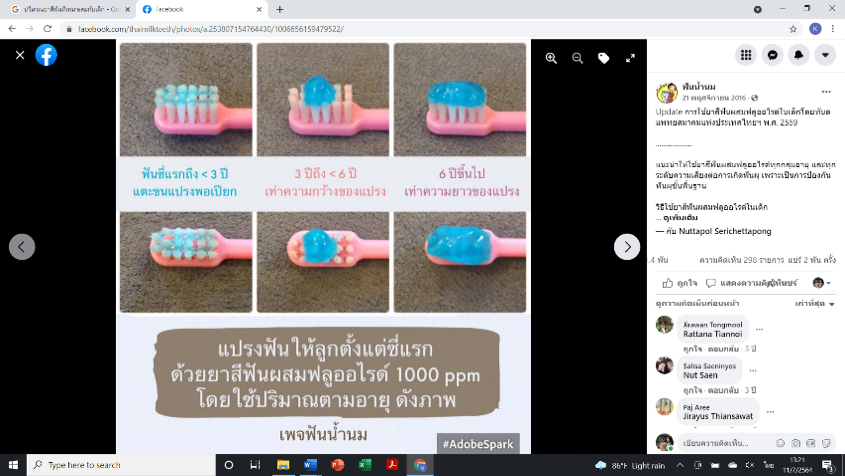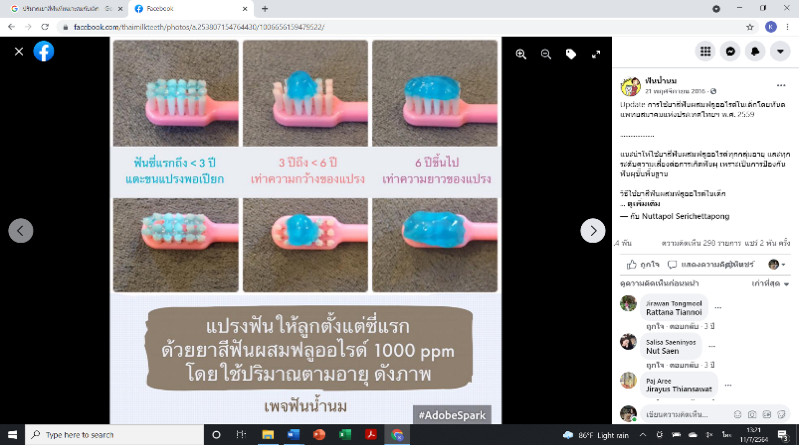 \| 2 🞏 ความกว้างแปรง  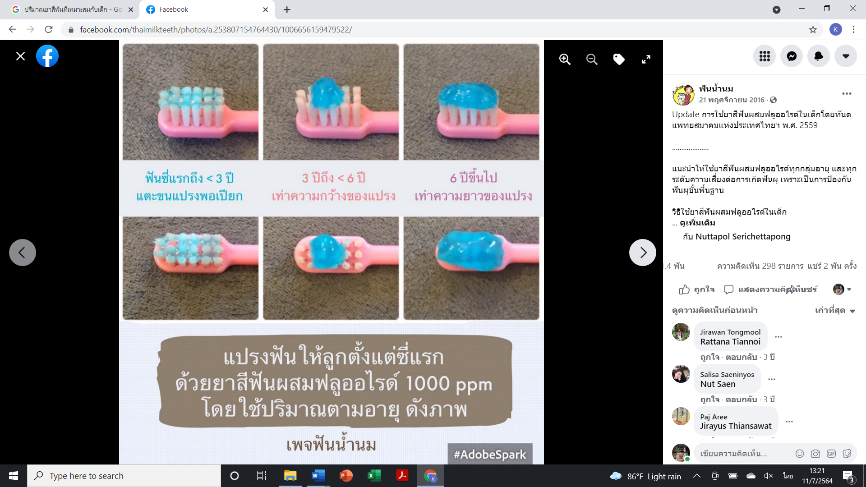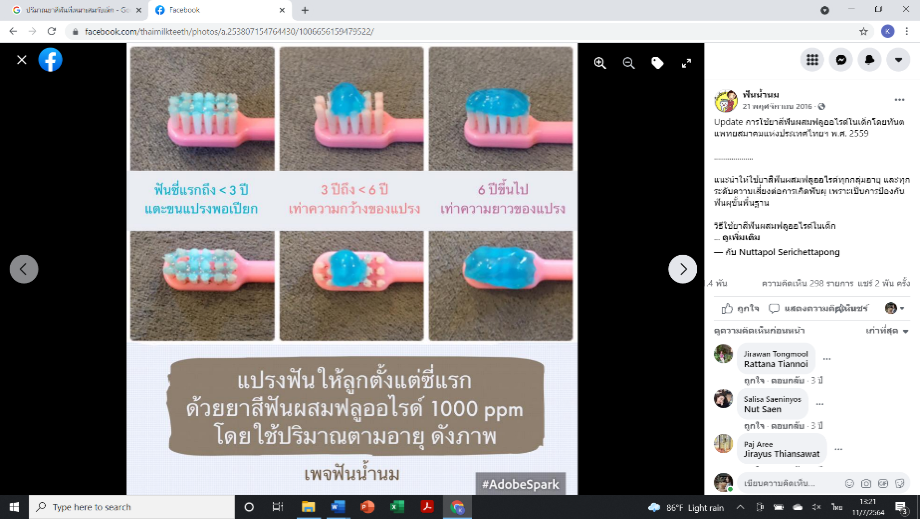 \| 3 🞏 ความยาวแปรง  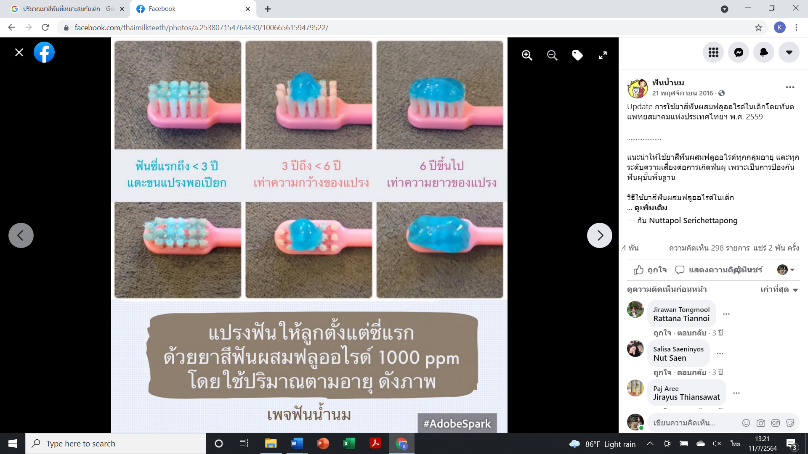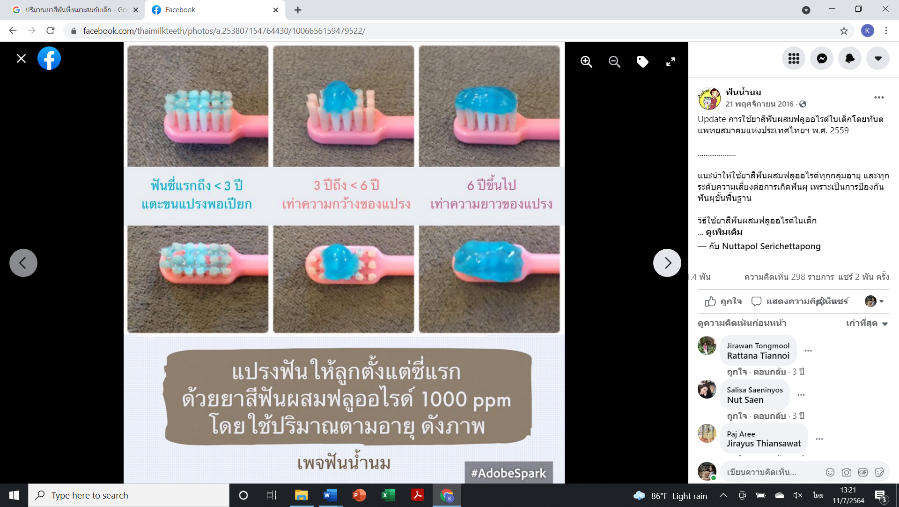 \| \| --- \| --- \| --- \| |

**3. ทัศนคติการดูแลสุขภาพช่องปาก**

คุณ...เห็นด้วยกับข้อความต่อไปนี้หรือไม่

ถ้า เห็นด้วยให้ตอบว่า เห็นด้วย ถ้าไม่เห็นด้วย ตอบว่า ไม่เห็นด้วย แต่ถ้าไม่แน่ใจ ให้ตอบว่า ไม่แน่ใจ

| **ลำดับ** | **คำถาม** | **ความรู้สึก** | | | | |
| --- | --- | --- | --- | --- | --- | --- |
|  |  | **1**  **ไม่เห็นด้วยอย่างยิ่ง** | **2**  **ไม่เห็นด้วย** | **3**  **ไม่แน่ใจ** | **4**  **เห็นด้วย** | **5**  **เห็นด้วยอย่างยิ่ง** |
|  | เป็นเรื่องปกติที่เด็กๆจะมีฟันน้ำนมผุ |  |  |  |  |  |
|  | ฟันผุเป็นโรคแค่ที่ฟัน ไม่มีผลเสียต่อส่วนอื่นๆ ของร่างกาย |  |  |  |  |  |
|  | ฟันน้ำนมผุไม่เป็นไร ไม่นานฟันแท้ก็มาแทนที่ ไม่มีผลเสียใดๆ |  |  |  |  |  |
|  | แม้ว่าเด็กจะฟันผุเยอะ เด็กก็ยังมีความสุขได้ (มีความสุขตามประสาเด็ก) |  |  |  |  |  |
|  | หากเด็กปวดฟัน เพราะฟันผุ เด็กจะงอแง เลี้ยงยากขึ้นกว่าเดิม |  |  |  |  |  |
|  | ลูกของคุณ...มีช่องปากที่สะอาดอยู่แล้วโดยคุณ...ไม่ต้องแปรงฟันให้ลูก |  |  |  |  |  |
|  | หากเด็กกินเฉพาะนม หลังจากกินนมแล้ว (ทั้งนมแม่ นมผง และนมอื่นๆ) ไม่จำเป็นต้องแปรงฟัน |  |  |  |  |  |
|  | การกล่อมให้ลูกนอนด้วยการกินนม เด็กจะหลับง่าย |  |  |  |  |  |
|  | หากคุณ...แปรงฟันให้ลูกทุกวัน จะป้องกันฟันผุได้ |  |  |  |  |  |
|  | เด็กๆ ที่มีฟัน 1-2 ซี่ ยังไม่ต้องแปรงฟันก่อนก็ได้ |  |  |  |  |  |
|  | การแปรงฟันให้ลูกตั้งแต่ฟันซี่แรกขึ้น จะทำให้การแปรงฟันในเด็กง่ายขึ้น เมื่อโตขึ้นมา |  |  |  |  |  |
|  | ถ้าวันไหนคุณ...ไม่มีเวลา หรือลูกร้องเยอะ ก็สามารถงดการแปรงฟันไปก่อนได้ |  |  |  |  |  |
|  | ถ้าคุณ...แปรงฟันแล้วลูกเหงือกลูกมีเลือดออก คุณ...จะหยุดแปรงทันที เพราะลูกต้องเจ็บ |  |  |  |  |  |
|  | การแปรงฟันในเด็กเล็กเป็นเรื่องที่ยากเกินกว่าที่คุณ...จะทำได้ |  |  |  |  |  |
|  | คุณ...สามารถแปรงฟันฟันให้ลูกได้ ถึงแม้ว่าลูกจะร้องจนคนอื่นบอกให้หยุดแปรง |  |  |  |  |  |
|  | คุณ...สามารถแปรงฟันให้ลูกได้ แม้ว่าลูกจะร้องไห้เยอะ และไม่ยอมให้แปรงก็ตาม |  |  |  |  |  |
|  | คุณ...จะแปรงฟันให้ลูกได้ จากการทำตามคำแนะนำในคลิปวิดีโอ |  |  |  |  |  |
|  | คุณ...ดูไม่ออก ว่าแปรงฟันเสร็จแล้วฟันสะอาดหรือไม่ เพราะขี้ฟัน และฟันเป็นสีขาวเหมือนๆ กัน และไม่รู้วิธีดูความสะอาด ว่าดูอย่างไร |  |  |  |  |  |
|  | คุณ...มีความตั้งใจในการแปรงฟันให้ลูกอย่างเต็มที่ |  |  |  |  |  |
|  | คุณ...สามารถให้คำแนะนำคนอื่นในการแปรงฟันให้ลูกได้ |  |  |  |  |  |

**4. ความรู้ในการดูแลสุขภาพช่องปาก**

คุณ...คิดว่า ข้อความต่อไปนี้ กล่าวได้ถูกต้องหรือไม่ ให้เลือก**ใช่** หรือ**ไม่ใช่** หากไม่ทราบ ให้เลือก**ไม่ทราบ**

| **ข้อที่** | **คำถาม** | **คำตอบ** | | |
| --- | --- | --- | --- | --- |
|  |  | **ใช่**  **(1)** | **ไม่ใช่**  **(2)** | **ไม่ทราบ**  **(3)** |
|  | ถ้าฟันน้ำนมผุมาก จะส่งผลให้ฟันดำ ยิ้มไม่สวยงามเพียงอย่างเดียว ไม่ได้ส่งผลอื่นๆ ต่อเด็ก |  |  |  |
|  | เด็กควรได้รับการแปรงฟันครั้งแรก เมื่อมีฟันกรามด้านที่ใช้เคี้ยวอาหารงอกขึ้นมาแล้ว |  |  |  |
|  | เด็กอายุน้อยกว่า 2 ขวบ ต้องแปรงฟันทุกวัน |  |  |  |
|  | เด็กอายุน้อยกว่า 2 ขวบ ควรแปรงฟันอย่างน้อยวันละ 1 ครั้ง |  |  |  |
|  | การเลือกแปรงสีฟันในเด็ก ควรเลือกแปรงขนแข็ง เพื่อสามารถขจัดขี้ฟันได้หมด |  |  |  |
|  | แปรงสีฟันที่ใช้ในเด็กเล็ก ใช้ขนาดเดียวกับเด็กโตได้ |  |  |  |
|  | วิธีแปรงฟันที่เหมาะสมในเด็กเล็ก คือ แปรงขึ้นแปรงลง |  |  |  |
|  | หากคุณเป็นคนแปรงฟันให้เด็ก ท่าแปรงฟันที่จะทำให้มองเห็นช่องปากชัดเจนที่สุด คือ ท่านอน |  |  |  |
|  | ขณะที่คุณแปรงฟันให้เด็ก การแหวกแก้ม จะทำให้เห็นฟันที่จะแปรงชัดขึ้น |  |  |  |
|  | ในเด็กเล็ก ไม่จำเป็นต้องเช็ดฟองออกทุกครั้งที่แปรงฟัน |  |  |  |
|  | เด็กสามารถใช้ยาสีฟันผู้ใหญ่ ที่มีส่วนผสมของฟลูออไรด์ได้โดยไม่เป็นอันตรายต่อสุขภาพ (ถ้าไม่นับเรื่องเด็กจะแสบปาก) |  |  |  |
|  | หากเด็กยังบ้วนน้ำไม่เป็น ควรเลือกใช้ยาสีฟันไม่มีฟลูออไรด์ |  |  |  |
|  | ถ้าลูกร้อง ดิ้นไปมาตอนแปรงฟัน ไม่ควรไปจับให้เด็กอยู่นิ่ง เพราะเด็กจะยิ่งต่อต้าน |  |  |  |
|  | สิ่งที่สำคัญที่สุดในการจัดท่าทางในการแปรงฟันในเด็ก คือ ศีรษะเด็กต้องอยู่นิ่ง และผู้แปรงต้องมองเห็นฟันที่จะแปรงชัดเจน |  |  |  |
|  | การจัดท่าทางในการแปรงฟันที่ถูกต้อง จะไม่ได้ทำให้เด็กรู้สึกเจ็บ |  |  |  |

**5.** **แบบประเมินความพึงพอใจสื่อ**

**ให้คุณ...เลือกข้อที่ตรงกับความคิดของคุณ... เพียง 1 ข้อ**

| 1. ความยาวในการพูดคุยในแต่ละวันมีความเหมาะสม  \| 1 🞏 ยาวไป \| 2 🞏 เหมาะสม \| 3 🞏 สั้นไป \| \| --- \| --- \| --- \| |
| --- | --- | --- | --- |
| 1. จำนวนวันที่ส่งข้อความ (30 วัน) มากไป น้อยไปหรือเหมาะสมแล้ว  \| 1 🞏 มากไป \| 2 🞏 เหมาะสม \| 3 🞏 น้อยไป \| \| --- \| --- \| --- \| |

**คุณ...มีความรู้สึกพึงพอใจต่อสื่อ Chatbot 30 วันฟันดี ในประเด็นต่อไปนี้ เพียงใด โดยมีคะแนน 5 ระดับ
ระดับ 5 หมายถึงพึงพอใจมากที่สุด 4 พึงพอใจอย่างมาก 3 พึงพอใจปานกลาง 2 พึงพอใจน้อย และ 1 พึงพอใจน้อยที่สุด**

| **ข้อที่** | **คำถาม** | **ความพึงพอใจ** | | | | |
| --- | --- | --- | --- | --- | --- | --- |
|  |  | **1**  พึงพอใจน้อยที่สุด | **2**  พึงพอใจน้อย | **3**  พึงพอใจปานกลาง | **4**  พึงพอใจอย่างมาก | **5**  พึงพอใจมากที่สุด |
|  | เข้าไปใช้หรือเล่น ได้ไม่ยาก |  |  |  |  |  |
|  | สื่อ เช่น ข้อความ วิดีโอ รูปภาพเข้าใจง่าย |  |  |  |  |  |
|  | ข้อมูลมีความน่าเชื่อถือ |  |  |  |  |  |
|  | พูดคุยภาษาที่เป็นกันเอง เข้าใจง่าย |  |  |  |  |  |
|  | เวลาคุณเล่น Chatbot แล้ว รู้สึกเหมือนได้คุยกับหมอจริงๆ |  |  |  |  |  |
|  | มีการให้กำลังใจในการดูแลลูกหลาน |  |  |  |  |  |
|  | การเรียงลำดับของเนื้อหาเหมาะสม ทำให้เข้าใจง่าย |  |  |  |  |  |
|  | ระยะเวลารอการโต้ตอบในแต่ละข้อความของ Chatbot ที่ไม่นานเกินไป |  |  |  |  |  |
|  | ระบบส่งข้อความต่อเนื่อง สม่ำเสมอทุกวัน และได้รับข้อความครบ 30 วัน |  |  |  |  |  |
|  | Chatbot ทำให้คุณ...อยากไปแปรงฟันให้เด็ก |  |  |  |  |  |
|  | คุณ...สามารถแปรงฟันให้เด็กได้จากการดูภาพ (Infographic) ใน Chatbot |  |  |  |  |  |
|  | Chatbot ทำให้คุณ...มั่นใจในการไปแปรงฟันให้เด็กได้จริง |  |  |  |  |  |
|  | ข้อมูลที่ได้รับจาก Chatbot มีประโยชน์ |  |  |  |  |  |
|  | ข้อมูลจาก Chatbot ทำให้คุณสามารถนำไปใช้ในชีวิตประจำวันได้จริง |  |  |  |  |  |
|  | ความพึงพอใจในภาพรวม |  |  |  |  |  |

| **คำถามปลายเปิด** |
| --- |
| 1. คุณ...มีความรู้สึก**พึงพอใจใน**ประเด็นอื่นๆอีกหรือไม่ สามารถบอกได้  - เนื้อหา   .........................................................................................................................  .........................................................................................................................   - การออกแบบ โครงสร้าง หรือระบบของ Chatbot เช่น ความสะดวกในการเข้าใช้งาน การส่งข้อความมาเวลาเดิมทุกวัน การพูดคุยที่เป็นธรรมชาติ มีการบันทึก การติดตามผล หรือการออกแบบที่ดึงดูดใจ   .........................................................................................................................  ......................................................................................................................... |
| 1. คุณ...มีความรู้สึก**ไม่พึงพอใจ**ต่อ Chatbot หรืออยากให้ปรับปรุง ในเรื่องใดบ้าง  - เนื้อหา   ........................................................................................................................  .........................................................................................................................   - การออกแบบ โครงสร้าง หรือระบบของ Chatbot เช่น เข้าใช้งานยาก ข้อความขาดหาย ไม่ได้ส่งมาทุกวัน พูดคุยแบบไม่เป็นธรรมชาติ หรือการออกแบบที่ไม่ดึงดูดใจ   ........................................................................................................................  ........................................................................................................................   - สิ่งที่ควรปรับปรุงใน Chatbot 30 วันฟันดี.......................................................................................................................   ....................................................................................................................... |
| 1. สาเหตุที่หยุดเล่น Chatbot 30 วันฟันดี   .......................................................................................................................  ..................................................................................................................... |
